# The effects of *Lactobacillus* and/or *Bifidobacterium* in fermented foods on cognitive health: a systematic review

**DOI:** 10.3389/fnut.2025.1682419

**Published:** 2025-12-03

**Authors:** Hayriye Sebnem Harsa, Carmen María González Domenech, Milica Prvulović, Zeynep Agirbasli, Erfan Bagherzadehsurbagh, Valentina Simeunović, Eleni Naziri, Elizabeth Adesemoye, Aycan Yigit Cinar, Arghya Mukherjee, Marta Laranjo, Bojana Vidović, Emilia Alves, Anđela Vukojević, Sine Özmen Toğay, Gamze Düven, Helen Saar, Seppo Salminen, Antonia Matalas, Diana Paveljšek, Else Schneider, Timur Liwinski, Christophe Chassard, Guy Vergères, Cornelia Bär, Smilja Praćer

**Affiliations:** 1 Department of Food Engineering, Faculty of Engineering, İzmir Institute of Technology, İzmir, Türkiye; 2 Department of Microbiology, School of Medicine, University of Malaga, Málaga, Spain; 3 Department of Neurobiology, Institute for Biological Research “Sinisa Stankovic”, National Institute of Republic of Serbia, University of Belgrade, Belgrade, Serbia; 4 Department of Food Engineering, Institute of Natural and Applied Sciences, Akdeniz University, Antalya, Türkiye; 5 Department of Food Science and Nutrition, School of Environment, University of the Aegean, Lemnos, Greece; 6 Department of Microbiology, Faculty of Life Sciences, Federal University Oye Ekiti, Ekiti State, Nigeria; 7 Department of Food Engineering, Faculty of Engineering and Natural Sciences, Bursa Technical University, Bursa, Türkiye; 8 Department of Food Biosciences, Teagasc Food Research Centre, Fermoy, County Cork, Ireland; 9 APC Microbiome Ireland, Cork, County Cork, Ireland; 10 MED-Mediterranean Institute for Agriculture, Environment and Development & CHANGE-Global Change and Sustainability Institute, Departamento de Medicina Veterinária, Escola de Ciências e Tecnologia, Universidade de Évora, Évora, Portugal; 11 Department of Bromatology, Faculty of Pharmacy, University of Belgrade, Belgrade, Serbia; 12 CBIOS - Universidade Lusófona’s Research Center for Biosciences & Health Technologies, Lisbon, Portugal; 13 Department of Food Engineering, Faculty of Agriculture, Bursa Uludağ University, Bursa, Türkiye; 14 Milk and Dairy Technology Programme, Department of Food Processing, Karacabey Vocational School, Bursa Uludag University, Bursa, Türkiye; 15 AS TFTAK Research Services, Tallinn, Estonia; 16 Center for Food and Nutrition Research, University of Turku, Turku, Finland; 17 Department of Nutrition and Dietetics, School of Health Sciences and Education, Harokopio University, Athens, Greece; 18 University of Ljubljana, Biotechnical Faculty, Ljubljana, Slovenia; 19 Center for Affective, Stress and Sleep Disorders, University Psychiatric Clinics (UPK) Basel, Basel, Switzerland; 20 Experimental Cognitive and Clinical Affective Neuroscience (ECAN) Laboratory, Department of Clinical Research (DKF), University of Basel, Basel, Switzerland; 21 UCA, INRAE, VetAgro Sup, UMRF, Aurillac, France; 22 Strategic Research Division Food Microbial Systems, Functional Nutritional Biology, Agroscope, Bern, Switzerland; 23 Competence Division Methods Development and Analytics, Biochemistry of Milk and Microorganisms, Agroscope, Bern, Switzerland

**Keywords:** fermented food, episodic memory, EFSA, functional food, bioactive metabolites, psychobiotics, gut-brain axis, microbiota

## Abstract

**Background:**

Psychobiotics are microorganisms that modulate brain function via the gut–brain axis and are increasingly studied for their cognitive benefits. *Lactobacillus* and *Bifidobacterium* species, widely present in fermented foods, are considered safe and may influence cognition by modulating neuroinflammation, neurotransmitters, and gut barrier integrity. This systematic review examined the effects of foods fermented with these species on cognitive performance in healthy adults and individuals with mild cognitive impairment.

**Methods:**

We conducted the systematic review following EFSA guidelines, Cochrane methodology, and a PROSPERO protocol, using CADIMA for study selection and data extraction. PubMed, Scopus, and Cochrane Library were searched (1 January 1970–31 August 2023) for human intervention and observational studies assessing cognitive outcomes after ingestion of foods fermented with *Lactobacillus* or *Bifidobacterium*. Eligible populations included healthy adults and individuals with mild cognitive impairment; studies involving disease were excluded. Screening, data extraction, and bias assessment followed Muka et al.’s 24-step guide using ROBINS and Cochrane/CADIMA frameworks. Evidence was synthesized narratively, while a non-systematic component examined food characteristics, potential mechanisms, and factors affecting bioavailability of bioactive constituents.

**Results:**

We included 21 studies (8 interventional, 13 observational). The majority of studies reported benefits, particularly in episodic memory, executive functions, and global cognition, but evidence was limited by inadequate controls, small sample sizes, short interventions, inconsistent domain assessment, and incomplete food characterization. Observational studies had larger populations and longer follow-ups but were limited by exposure assessment and depth of cognitive testing.

**Conclusion:**

Consumption of foods fermented with *Lactobacillus* and/or *Bifidobacterium* species may offer promising cognitive benefits. However, following EFSA’s guidance on the substantiation of health claims, the current evidence is “neither convincing nor sufficient” to establish a causal relationship. Well-designed studies with thorough product characterization are needed to substantiate effects and support potential health claims.

**Systematic review registration:**

This study was registered at the Open Science Framework (10.17605/OSF.IO/Z6GRW).

## Graphical abstract

**Figure:**
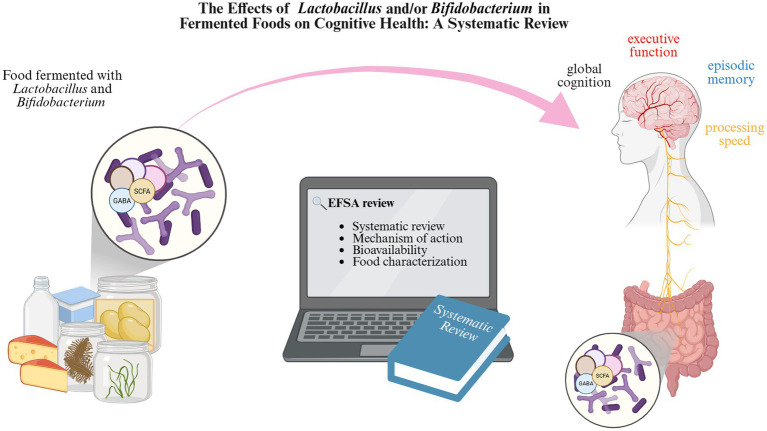

## 1 Introduction

Cognitive function is a critical determinant of overall well-being, influencing daily activities, productivity, and quality of life. Emerging evidence suggests that dietary factors play a significant role in cognitive health, with particular interest in the gut-brain axis as a key mediator (1–4). Fermented foods (FFs), especially those rich in bacteria *Lactobacillus* and *Bifidobacterium* (*L&B*), have garnered attention for their potential neuroprotective effects (5). They are often used as starter cultures in FFs such as yogurt, kefir, kimchi and tempeh. The safety and health-promoting properties of these probiotic microorganisms are well documented, including their effects on gut and brain function (6, 7). In addition to the live microbes, their metabolic by-products, such as short-chain fatty acids (SCFAs), organic acids and bioactive peptides, contribute to host health, including gut-brain signaling pathways (8, 9). The cognitive benefits of these bacteria are thought to be mediated by multiple mechanisms. *L&B* can positively modulate the gut microbiota and support microbial balance (10, 11), improve gut barrier function and reduce inflammation (7), and influence neurotransmitter levels (12). Some *Lactobacillus* strains are able to produce *γ*-aminobutyric acid (GABA), an important inhibitory neurotransmitter involved in cognition and mood (13). In addition, *B. infantis* has been shown to modulate the hypothalamic–pituitary–adrenal axis (HPA axis), reducing stress responses associated with cognitive decline (14).

FFs and beverages have long been associated with various health benefits, including potential effects on brain and cognitive health, as evidenced by both preclinical and clinical results (15, 16). European Food Safety Authority (EFSA) has systematically rejected a wide range of health claims related to FFs, e.g., numerous claims on fermented whey, probiotics (17), fermented skimmed milk for claims on immune function in children (18) and probiotic dairy products for claims on gut and immune health (19, 20). To date, the only claim authorized by EFSA that relates to FFs is for yogurt and fermented milk that contribute to improved lactose digestion (21). No health claims linking FFs to cognitive function have been approved and a clear cause-effect relationship in this area remains unproven. Recent systematic reviews have provided preliminary evidence that probiotic supplementation involving specific, standardized strains and FFs containing live microbes along with other bioactive compounds can improve cognitive function, stress regulation and mood in both healthy and clinical populations (22–27). Most studies included in these reviews examine both probiotic supplements and FFs rather than FFs exclusively, resulting in heterogeneous findings, and strains responsible for the effects as well as the corresponding mechanism of action have not yet been elucidated (26, 28). Despite the increasing interest in the impact of *L&B* in gut–brain axis mechanisms, there is a lack of comprehensive and systematic evidence on the cognitive effects of *L&B* in FFs, as the variations in study design, sample populations, intervention protocols and magnitude and physiological relevance of outcome warrant a structured and comprehensive synthesis of existing evidence (27). This review therefore aims to identify and clarify the evidence gaps that have typically led to EFSA rejections in order to provide a clearer basis for future substantiated claims in the cognitive domain.

Therefore, this systematic review, conducted within the framework of COST Action CA20128 – PIMENTO (Working Group 3), aims to critically evaluate the association between the consumption of *Lactobacillus*- and *Bifidobacterium*-FFs and cognitive outcomes in healthy adults and individuals with mild cognitive impairment (MCI), using evidence from interventional studies (InSs) and observational studies (ObSs). In accordance with the guidelines set forth by the EFSA, “an increase, maintenance, or reduced loss of cognitive function in one or more of its domains is considered a beneficial physiological effect” (29) and we evaluated several domains related to cognition: episodic memory (verbal and visual), executive functions (attention, alertness, working memory, problem-solving), speed processing and global cognition (30). The review follows a registered study protocol^1^ and the structured methodology proposed by Muka et al. (31), which incorporates principles of Cochrane systematic reviews, including study selection, risk of bias (RoB) assessment and evidence synthesis (39). In line with EFSA’s scientific requirements for health claims (19, 20) the review also includes a non-systematic component addressing food characteristics, mechanisms of action, bioavailability and safety. This comprehensive approach provides new insights into practical, real-world nutritional strategies for cognitive health while highlighting important research gaps and methodological limitations.

## 2 Methods

### 2.1 Systematic review

#### 2.1.1 Study protocol

This systematic review was registered on the Open Science Framework (OSF) (32) on October 11th 2024 and conducted following established methodological standards, drawing on the Cochrane Handbook (33) and adhering to the updated PRISMA (Preferred Reporting Items for Systematic Reviews and Meta-Analyses) guidelines (34) for transparent and comprehensive reporting (Supplementary material 1). The design, coordination, progress, updating, and evidence summarization of the current systematic review were carried out according to steps outlined in Muka et al. (31).

Following the reclassification of the *Lactobacillaceae* family based on various genetic analyses (35), several former *Lactobacillus* species have been reassigned to newly established genera, such as *Lactiplantibacillus*, *Limosilactobacillus*, and *Lacticaseibacillus*. For clarity and consistency throughout this review, we collectively refer to these species as “*Lactobacillus*,” in contrast to *Bifidobacterium* species.

#### 2.1.2 Literature search

We conducted a systematic literature search in PubMed, Scopus, and the Cochrane Library for studies published in English between January 1, 1970, and August 31, 2023. Generic search terms developed by Alisa Berger (University Library Medicine, University of Zürich, Zürich, Switzerland) were previously published (39) and used to cover a broad range of FFs across all food groups, human studies, and dietary intake (Supplementary Table S1). Included studies were human InSs, human ObSs, and systematic reviews with or without meta-analyses to ensure comprehensive coverage.

#### 2.1.3 Population intervention control outcome (PICO) criteria

Exclusion and inclusion criteria were defined based on the research question of this review: Does consumption of foods fermented with *Lactobacillus* sp. and/or *Bifidobacterium* sp. have a beneficial effect on cognitive performance in a healthy adult population including mild cognitive impairment? All details are listed in Table 1. Defining appropriate controls remains a major challenge in nutritional science. To identify research gaps and gather comprehensive data from human studies, we followed EFSA guidance and included all relevant studies, regardless of control quality. This is the reason why we do not have exclusion criteria for control. For studies meeting the population, intervention, and outcome (PIO) criteria, control quality was subsequently assessed and classified.

**Table 1:** PICO criteria.

Criteria	Inclusion	Exclusion
Population	Healthy adults (≥18 years) Mild cognitive impairment, Metabolic syndrome Irritable bowel syndrome, Prediabetes, Obesity Eating disorders, Pregnancy On medication	Any disease, including neurological or psychiatric diseases
Intervention	Foods fermented with *L&B*: dairy, meat, fish, fruits and vegetables, beverages, legumes, cereals, grains, and FF extracts or powders, with no restrictions on dose or duration Probiotics introduced at the beginning of the fermentation process	Alcoholic beverages with more than 1.25% alcohol Addition of probiotics after fermentation process Addition of prebiotic fibers Addition of bioactive compounds
Control	Ideal control Fermented product identical to the intervention in appearance, taste, texture, raw material, processing, added ingredients, and storage, but without the active *L&B* components High-quality control Non-fermented products matching the intervention in nutrient profile and appearance, taste, texture, raw material, processing, added ingredients, and storage conditions Low-quality control Non-fermented product that did not match the intervention in nutrient profile, appearance, taste, texture, raw material, processing, added ingredients, and storage conditions	
Outcome	General cognitive ability Language and communication, Verbal and visual memory Long- and short-term memory, Attention Alertness (increased performance in reaction time or speed of response) Problem solving Processing speed Executive function Dementia risk	Mood Stress Anxiety Sleep Alertness (specific mood/affect construct such as ‘feeling alert’) Enthusiasm Calmness Confusion Depression Fatigue Tension Distress Studies assessing cognition only *via* functional magnetic resonance imaging (fMRI)

In order to assess the quality of control, we classified three types of controls:

1. Ideal control was set to be a fermented product identical to the intervention in appearance, taste, texture, raw material, processing, added ingredients, and storage, but without the active *L&B* components. No study included an ideal control.
2. High-quality control was non-fermented products matching the intervention in nutrient profile and all other characteristics listed above.
3. Low-quality control was a non-fermented product that did not match the intervention in one or more of the listed characteristics. ObSs were included if all PIO criteria were met, regardless of control quality. In these cases, controls were typically defined as no (placebo) or lower intake of the FF in question or consumption of a non-fermented equivalent.

The impact of consuming foods fermented with *L&B* on cognitive performance was assessed through human studies using tests across various cognitive domains (36, 37), including dementia risk as a measure of cognitive health. We excluded studies focused solely on mental health domains.

##### 2.1.3.1 Data extraction and analysis

**Figure 1:**
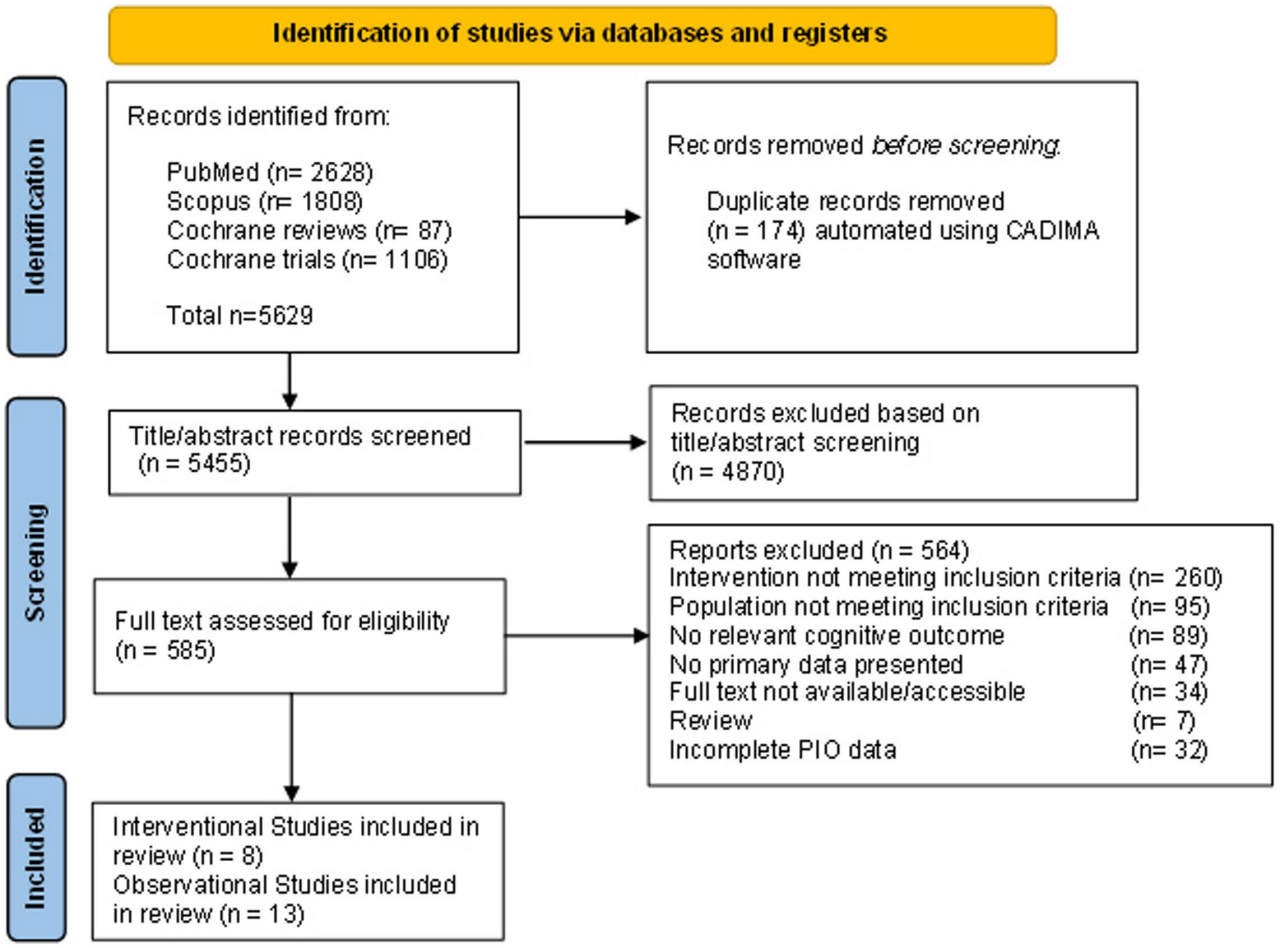
Flow diagram illustrating the identification of studies for inclusion.

We used the CADIMA (Central Access Database for Impact Monitoring and Assessment) web tool for the study selection process (38) (Figure 1). After eliminating the duplicates, 19 co-authors conducted a training session using a consistency test in CADIMA. The final study selection process included title and abstract screening, followed by full-text screening, with each study evaluated independently by 8 pairs of reviewers, while 3 reviewers resolved inconsistencies. The study selection was based on the predefined inclusion and exclusion criteria based on the PIO framework to ensure a systematic and transparent approach. Discrepancies at any stage were resolved through discussion, and/or a third reviewer was consulted. During the process of collection, comparison, and selection of references for retrieval, we also performed searches for additional references using the reviews obtained during the literature search (up to 31st of December 2024). The final selection included 21 articles, 13 ObSs and 8 InSs.

To extract the data contained in the included studies, we used a predefined data collection form in Excel (Supplementary Table S2). Before the final data extraction process, we performed a training session for extracting data from studies, using one Randomized Controlled Trials (RCTs) as an InS and one ObS as templates, improving the content of the data extraction (DE) table and instructing the reviewers on best practice in extracting the data. Eight independent pairs of reviewers extracted detailed data from each article. After merging all data and resolving inconsistencies, a final database for descriptive analysis was created.

##### 2.1.3.2 Quality and bias of study (Q&B)

If control quality was found to be sufficient (see section 17, Study Protocol (39)) we performed Quality and bias of study (Q&B) evaluation in those articles. If not, Q&B was not performed. The evaluation of control and Q&B was performed independently by two reviewers for each study. For RCTs, we used the Revised Cochrane RoB tool for randomized trials (RoB 2) (40), evaluating five domains: randomization, deviations from intended interventions, missing data, measurement of outcomes, and selection of the reported result. Each trial was rated as having low, some concerns, or high RoB on those domains.

#### 2.1.4 Data synthesis

In this systematic review, the quality of evidence was evaluated using a grading system based on the EFSA steps for “Substantiation of a causal relationship between consumption of the fermented food and the functional effect” and “Characterization of the relationship between consumption of the fermented food and functional effect.”

### 2.2 Non-systematic part of the review

#### 2.2.1 Supportive evidence-mechanism of action and bioavailability

Information on the FFs used in the included studies was extracted and summarized in line with the “Bioavailability” section of the Scientific and Technical Guidance for the Preparation and Presentation of a Health Claim Application (Revision 3, 2021), following the workflow detailed in the Supplementary materials.

Data on bioavailability (including bioaccessibility) and mechanisms of action (including the gut microbiota’s role in cognition) were systematically collected according to Section 5.2.3, ‘Supportive Evidence – Bioavailability and Mechanism(s) of Action. To establish biological plausibility, a structured approach was applied based on three key areas: (1) identification and characterization of bioactive compounds, (2) interaction with the gastrointestinal tract, and (3) systemic and cognitive effects.

Extracted information for each study is presented in the “Bioavailability” and “Mechanism of Action” sections of this review.

#### 2.2.2 Characteristics of fermented foods included in the studies

The information on the characteristics of the FFs employed in the studies was extracted and summarized in accordance with the section “Characterization of the food/constituent” in the “Scientific and technical guidance for the preparation and presentation of the health claim application” (Revision 3, 2021), following the workflow presented in Supplementary material. The information for each individual study was then summarized and presented in the section “Characterization of the food/constituent” according to the food group.

## 3 Results

In this systematic review, we aimed to assess evidence on FFs and cognitive function using the EFSA framework for health claims as structured by Muka et al. (31). Using a systematic approach to identify relevant literature, we included 21 eligible studies. In accordance with EFSA guidance, we also assessed product characterization, bioavailability and mechanisms of action to determine whether the available evidence met the criteria normally required for the scientific evaluation of health claims.

We included 8 InSs: 6 classic randomized, double-blind, placebo-controlled, parallel-group studies, one randomized, controlled crossover study and one controlled study without explicit randomization. The majority of the InSs were conducted in Asia: 4 from South Korea, one from Japan, Indonesia, USA, and the UK representing the only European country (Table 2).

**Table 2:** Type of studies and population characteristics.

Author, year	Title of the paper	Type of study	Cohort	Population characteristics
*Interventional studies*				
Reid et al. (41)	The Effects of Fermented *Laminaria japonica* on Short-Term Working Memory and Physical Fitness in the Elderly	Interventional randomized, double-blind, placebo-controlled, parallel-group studies	NA	*n* = 40 adults finished the trial Age: intervention group 72.35 ± 5.54 yrs. placebo 74.57 ± 5.69 yrs. Gender: NI BMI: NI, but weight and height were reported Obesity: NI
Hwang et al. (46)	Efficacy and Safety of *Lactobacillus plantarum* C29-Fermented Soybean (DW2009) in Individuals with Mild Cognitive Impairment: A 12-Week, Multi-Center, Randomized, Double-Blind, Placebo-Controlled Clinical Trial	Interventional randomized, double-blind, placebo-controlled, parallel-group studies	NA	*n* = 92 Age: Intervention:68.0 ± 5.12 yrs. Placebo: 69.2 ± 7.00 yrs. Gender: men and women MCI BMI: 23.9–24.6 kg/m^2^ Obesity: NI
Ohsawa et al. (42)	*Lactobacillus helveticus*-fermented milk containing lactononadecapeptide (NIPPLTQTPVVVPPFLQPE) improves cognitive function in healthy middle-aged adults: a randomized, double-blind, placebo-controlled trial	Interventional randomized, double-blind, placebo-controlled, parallel-group studies	NA	*n* = 61 Age: 50–70 yrs. Gender: men and women BMI/Obesity: NI
Handajani et al. (45)	Tempeh Consumption and Cognitive Improvement in Mild Cognitive Impairment.	Interventional controlled trial without explicit randomization	NA	*n* = 84 Age 60–70 yrs. Gender: men and women MCI BMI/Obesity: NI
Cannavale et al. (48)	Consumption of a fermented dairy beverage improves hippocampal-dependent relational memory in a randomized, controlled cross-over trial	Interventional randomized, controlled crossover study	NA	*n* = 24 Age: 25–45 yrs. Gender: men and women BMI/Obesity: NI
Benton et al. (47)	Impact of consuming a milk drink containing a probiotic on mood and cognition	Interventional randomized, double-blind, placebo-controlled, parallel-group studies	NA	*n* = 126 Age 48–79 yrs. Gender: men and women BMI/Obesity: NI
Chung et al. (43)	Fermented milk of *Lactobacillus helveticus* IDCC3801 improves cognitive functioning during cognitive fatigue tests in healthy older adults	Interventional randomized, double-blind, placebo-controlled, parallel-group studies	NA	*n* = 36 Age: 60–75 yrs. Gender: men and women BMI: 24.85 ± 3.05 kg/m^2^
Park et al. (44)	A randomized, double-blind, placebo-controlled study on the memory enhancing effect of Lactobacillus fermented *Saccharina japonica* extract,	Interventional randomized, double-blind, placebo-controlled, parallel-group studies	NHANES, USA	*n* = 69 Age 18–65 yrs. Gender: men and women BMI/Obesity: NI
*Observational studies*				
Park et al. (56)	The association between dairy product consumption and cognitive function in the National Health and Nutrition Examination Survey	Cross-sectional studies	NHANES, USA	Cohort 1 *n* = 4,355 Age: 20–59 yrs. *n* = 4,282 Cohort 2 *n* = 2,189 Age: ≥ 60 yrs. Gender: men and women BMI/Obesity: BMI included, but not reported
Han et al. (62)	The Relationship Between Fermented Dairy Consumption with Cognitive Function Among Older US Adults	Observational Cross-sectional studies	NHANES, USA	*n* = 2,462 Age: > 60 yrs. Gender: men and women BMI/Obesity: 28-36 kg/m^2^
Ylilauri et al. (61)	Associations of dairy, meat, and fish intakes with risk of incident dementia and with cognitive performance: the Kuopio Ischaemic Heart Disease Risk Factor Study (KIHD).	Observational Baseline cohort study	Kuopio Ischaemic Heart Disease Risk Factor Study (KIHD)	*n* = 3,235 Age: 53 ± 5.1 yrs. Gender: men BMI: 26.9 ± 3.6 kg/m^2^
Kesse-Guyot et al. (53)	Consumption of Dairy Products and Cognitive Functioning: Findings from the SU. VI. MAX 2 Study	Observational Follow-up of an RCT	SU. VI. MAX and SU. VI. MAX 2 cohort studies, France	*n* = 3,076 Age: 65.5 yrs. Gender: men and women BMI: 24.4 ± 3.4 kg/m^2^
Ni et al. (52)	Dairy Product Consumption and Changes in Cognitive Performance: Two-Year Analysis of the PREDIMED-Plus Cohort	Observational Prospective cohort study	PREDIMED-Plus study cohort, Spain	*n* = 4,668 Age: 55–75 yrs. Gender: men and women BMI/Obesity: 27–40 Obesity included, BMI > 40 kg/m^2^ Inclusion: metabolic syndrome
Muñoz-Garach et al. (58)	Milk and Dairy Products Intake Is Related to Cognitive Impairment at Baseline in Predimed Plus Trial	Observational Cross-sectional studies	PREDIMED-PLUS study, Spain	*n* = 6,426 Age: 55–75 yrs. Gender: men and women BMI/Obesity: 27–40 Obesity included, BMI > 40 kg/m^2^ Inclusion: metabolic syndrome
Hogervorst et al. (57)	High Tofu Intake Is Associated with Worse Memory in Elderly Indonesian Men and Women	Observational Cross-sectional studies	Rural West and Central Java, and urban site Jakarta, Indonesia	*n* = 719 Age: 52–98 yrs. 65% of women Gender: men and women BMI/Obesity: NI
Hogervorst et al. (49)	Borobudur revisited: soy consumption may be associated with better recall in younger, but not in older, rural Indonesian elderly	Observational Cross-sectional studies	Central Java, Indonesia	*n* = 142 Age: 56–97 yrs. Women, 61% Gender: men and women BMI/Obesity: NI
Tessier et al. (50)	Milk, Yogurt, and Cheese Intake Is Positively Associated With Cognitive Executive Functions in Older Adults of the Canadian Longitudinal Study on Aging.	Observational Cross-sectional studies	Canadian Longitudinal Study on Aging (CLSA)	*n* = 7,945 participants Age: 65–86 yrs. Gender: men and women BMI/Obesity: 27.4–27.8 kg/m^2^ Obesity included
Kim et al. (60)	Inverse Association between Cheese Consumption and Lower Cognitive Function in Japanese Community-Dwelling Older Adults Based on a Cross-Sectional Study.	Observational Cross-sectional studies	Cohort 1: “The Otassha Study 2017 Cohort”, Cohort 2: volunteer participants from 18 neighborhoods near the Tokyo Metropolitan Institute of Gerontology, Itabashi, Tokyo, Japan	*n* = 1,504 Cohort 1: Age: 65–99 yrs. Gender: women Cohort 2: Age: 75–85 yrs. Gender: men and women BMI/Obesity: 22.70 ± 3.35 kg/m^2^
Suzuki et al. (59)	Association between the Intake/Type of Cheese and Cognitive Function in Community-Dwelling Older Women in Japan: A Cross-Sectional Cohort Study	Observational Cross-sectional cohort study	Japanese observational cohort of community-dwelling older women (“The Otassha Study”)	*n* = 1,035 Age: ≥ 65 yrs. Gender: women BMI/Obesity: NI
de Goeij et al. (54)	Associations between the Intake of Different Types of Dairy and Cognitive Performance in Dutch Older Adults: The B-PROOF Study	Observational Cross-sectional studies	B-PROOF study, Netherlands	*n* = 619 Age: ≥ 65 yrs. Gender: men and women BMI/Obesity: 27.1 ± 3.7
Ortega et al. (55)	Effect of dairy consumption on cognition in older adults: A population-based cohort study	Observational Population-based cohort study	CoLaus|PsyColaus cohort in Lausanne, Switzerland.	Age: >59 *n* = 6,734 Age: 35–75 yrs. Gender: men and women BMI/Obesity: % of participants with normal weight/overweight and obesity is reported

BMI: Body Mass Index; B-PROOF: B-vitamins for the PRevention of Osteoporotic Fractures; CLSA: Canadian Longitudinal Study on Aging; CoLaus: Cohorte Lausannoise; KIHD: Kuopio Ischaemic Heart Disease Risk Factor Study; MCI: Mild Cognitive Impairment; NA: Not Applicable; NHANES: National Health and Nutrition Examination Survey; NI: No Information; PREDIMED: PREvención con DIeta MEDiterránea (in English: Prevention with Mediterranean Diet); PsyColaus: Psychiatric arm of CoLaus; SU. VI. MAX: Supplementation en VItamines et Minéraux AntioXydants (in English: Supplementation with Antioxidant Vitamins and Minerals); RCT: Randomized Controlled Trial.

Of the 13 ObSs, 5 were cohort studies and the remaining 8 were cross-sectional studies (Table 2), and several used common or overlapping cohorts (Table 2 and Supplementary Table S3), with a wide geographical diversity including Asia, Europe and USA, increasing the global relevance of the results. Ethnicity was not explicitly reported in any of the InSs and ObSs but can be inferred from location.

### 3.1 Population

We have evaluated the study populations and their characteristics to determine whether the results can be extrapolated to the broader intended population. Across the included studies, the study populations varied in terms of health status, age, and sex (Table 2).

InSs involved modest sample sizes, generally with fewer than 50 participants in intervention arms (41–44) (Table 2). Three studies exceeded this range with *n* = 84 (45), *n* = 92 (46) and *n* = 126 (47). The lowest number of participants was *n* = 24 (48).

Sample sizes in ObSs varied significantly, ranging from several hundred to several thousand participants, supporting robust statistical analysis (Table 2). The smallest sample was *n* = 142 (49), while the largest was *n* = 7,945 (50).

Most of the InSs included participants aged 48–79 years, with mean ages typically in the 60s to early 70s (41–44) (Table 2). Park et al. (44) included a wider age range (18–65), though the mean age remained in the early 30s. Only Cannavale et al. (48) focused on a distinctly younger cohort (25–45 years) without clear demographic or epidemiological rationale, possibly reflecting an interest in early preventive strategies.

ObSs predominantly involved older adults, although the age range spanned from 20 to 99 years (Table 2). Most studies focused on individuals aged 55 and above (50, 52–54), with mean participant ages typically between 65 and 73 years. Ortega et al. (55) and Park et al. (56) included younger adults (35–75 and 20–99, respectively). These age profiles align with the cognitive focus of the research, reinforcing relevance to aging populations.

Across both InSs and ObSs, participant baseline characteristics and potential confounders were inconsistently reported and controlled and are summarized in Supplementary Table S4.

#### 3.1.1 Health

Health-related confounders in InSs were inconsistently addressed. Some studies considered supplement or nootropic intake, diabetes, FF intake, exercise, alcohol and sleep (41, 43, 45, 46). Benton et al. (47) reported on mental and physical health status at baseline. Only two studies provided Body Mass Index (BMI) data (43, 46), and few mentioned gastrointestinal problems (43, 48). Pregnancy and obesity were not addressed. InSs involved mostly cognitively healthy participants, with studies of Handajani et al. (45) and Hwang et al. (46), including only participants with MCI. Baseline cognition was assessed in all studies using validated cognitive tests.

The health assessment in ObSs varied greatly. Medication use was reported in 7 out of 13 studies, mostly by self-report. BMI was usually included, except in two studies (49, 57), while two studies focused on the same cohort on overweight/obese individuals (52, 58). Several studies excluded participants with extreme energy intake or poor nutritional status (50, 56). Pregnancy, eating disorders and diseases that affect nutrient intake were largely disregarded. Most ObSs included cognitively healthy, community-dwelling adults with cognitive status evaluated at baseline using the Mini-Mental State Examination (MMSE) (52, 54, 55, 58–61). Some studies (61, 62) relied on general cognitive or neurological assessments without reporting MMSE scores, while others did not report any baseline cognitive status (49, 57).

#### 3.1.2 Sex

Several InSs reported sex distribution. Handajani et al. (45) noted that 71.4% of participants were female and Benton et al. (47) had a predominantly female sample. Park et al. (44) included sex-balanced groups, and Cannavale et al. (48), Ohsawa et al. (42) and Chung et al. (43) included both sexes providing male to female ratios. None of the studies performed sex-stratified analyses, limiting insights into sex-based effects.

Sex distribution across all examined ObSs was often balanced, though not always reported. Only two studies were sex-specific focused exclusively on men (61) or on women (59).

Several ObSs (49, 50, 52, 54, 58–60) reported that the studies involved community-dwelling adults and only two studies focused on rural areas (49, 57). Analysis of the evidence and gaps regarding the studied population is presented in the Section 4.3.1.

**Table 8:** Overview of PICO evidence and gaps with product, mechanistic, and bioavailability information (EFSA Framework).

PICO criteria	Evidence	Gap
Population		
InS	Number of participants fewer than 50 per arm	Small sample sizes
	Healthy adults with mean age: 60s–70s Two studies included younger participants	Obesity not reported, BMI rarely Participants’ health status was self-reported with high variability.
	Exclusion criteria involved conditions or factors affecting cognition (diabetes, psychiatric disorders, alcohol use, medications)	Ethnicity, pregnancy, or eating disorders were not reported
	Gender distribution reported in most trials	Gender-specific analyses were not reported
ObS	Hundreds to thousands of community-dwelling adult participants	Only two examined the rural areas
	Healthy, aged 55 and older	Obesity was reported but was not analyzed by subgroups.
	Balanced gender representation	None conducted gender-specific analyses, or reported ethnicity, pregnancy, or eating disorders
Intervention		
InS	Intervention durations from 4 to 24 weeks	None included a follow-up phase
	Most used daily dosing regimens	Only *Lactobacillus* spp. used as treatment, *Bifidobacterium* spp. was only used in one study
	The majority focused on dairy-based FF	Only one study investigated dose–response None established minimal effective dose
	Most commonly assessed foods were fermented milk and yogurt, followed by soy-based FF (tempeh, fermented soy powder) and fermented seaweed powder.	Fermented vegetables, grains, meat and fish were not analyzed
	Microbial strains were specified	Only 4 studies reported microbial counts (e.g., colony-forming units, CFUs)
		The potential inaccuracy of dietary recall in cognitively impaired participants was rarely addressed
ObS	Most commonly assessed foods were fermented dairy (cheese and yogurt), two assessed soy-based FF	None of the studies reported microbial counts (e.g., colony-forming units, CFUs), or at least range
	Five included follow-ups, ranging from 2 to 22 years	The potential inaccuracy of dietary recall in cognitively impaired participants was rarely addressed
	Studies used FFQs to estimate habitual intake	
Control		
InS	Three studies had high-quality controls	None used the “ideal control”, majority of studies (5) had low-quality controls (sensory matching or had insufficient detail about the control content)
ObS	Three studies, although assessed as low-quality control, used non-fermented controls (distinguished the effects of fermentation and bacteria on cognitive performance in a free-living, large group population during a longer period of consummation and follow-up)	All studies had low-quality control
Outcome		
InS	Seven studies showed positive cognitive outcomes of consuming fermented food (both dairy-and plant- based)	Lack of follow-up to assess the persistence and duration of the effects
	The most common cognitive domain influenced by the FF was episodic memory (hippocampal-dependent), followed by executive functions and global cognition.	Lack of consistency in the selection of cognitive outcomes (majority of studies assessed three cognitive domains, the number varied considerably, with some evaluating only one and others more)
ObS	Ten studies showed positive associations between FF consumption and cognitive outcomes	Five studie**s** lacked domain-specific cognitive-depth, using only one cognitive test
	The most common cognitive domain influenced by the FF was episodic memory (hippocampal-dependent), followed by executive functions and global cognition.	High heterogeneity of tools used for cognitive assessment
Quality and Bias	RoB2 tool identified that two intervention studies had a low risk of bias, while one study raised some concerns.	Out of 21 InSs and ObSs, Q&B evaluation could only be performed for 3 studies.
Mechanism of Action	FFs serve as a dietary means to modulate cognitive health through the MGBA, leading to systemic benefits and improved brain function *via* beneficial changes in gut microbiota. FFs can directly or indirectly boost neuroactive compounds; for example, kefir enhances GABAergic and serotonergic signaling, improving memory and mood in mice. *Lactobacillus fermentum* A2.8 from tempeh produces GABA, likely contributing to its cognitive effects. FFs and their microbes/bioactive compounds mitigate chronic low-grade inflammation linked to cognitive decline. They reduce pro-inflammatory cytokines and improve gut barrier integrity, thus lowering neuroinflammation. FFs increase antioxidant enzyme activity (like SOD and CAT) and reduce oxidative stress, protecting neurons. Fermented seaweed is especially noted for these effects. FFs (yogurt, kefir, fermented seaweed) raise Brain-Derived Neurotrophic Factor (BDNF), vital for neurogenesis, synaptic plasticity, learning, and memory. LAB in FFs transform less bioavailable compounds, such as soy isoflavones, into more absorbable and active aglycones (daidzein, genistein, glycitein), which offer estrogen-like brain protection. FFs positively impact intestinal barrier integrity, with some *Lactobacillus* strains specifically helping preserve it, crucial for preventing neuroinflammation.	Information on potential mechanisms in human interventional and observational studies is scarce; this is the most significant gap. A clear picture of how individual *Lactobacillus* and *Bifidobacterium* strains, or their combinations, specifically contribute to cognitive improvements in humans is lacking. No studies specifically investigated cognitive effects from *Bifidobacterium*-fermented products alone; these are usually supplementary cultures. It’s hard to attribute cognitive effects solely to *Lactobacillus* and *Bifidobacterium* given the synergistic or independent roles of other microbes and transformed food components. Many detailed mechanistic findings (e.g., BDNF expression, neurotransmitter changes) come from animal models, necessitating more human studies for confirmation. The impact of genetic predisposition, pre-existing cognitive status, and baseline gut microbiota on FF effectiveness and mechanisms is not well-explored.
Bioavailability	Substantial evidence is found on bioavailability and bioactivity of various compounds in fermented dairy, soy, and brown algae. Fermentation, especially by *Lactobacillus*, significantly boosts compound bioavailability and bioactivity in dairy, soy, and seaweed. Soy fermentation converts less absorbable isoflavone glycosides into more bioavailable aglycones, which cross the blood–brain barrier for neuroprotection. Dairy fermentation releases bioactive “lactopeptides” (e.g., WY) and enriches products with GABA, influencing neurotransmission. Kelp fermentation cleaves fucoidan into absorbable low-molecular-weight fragments and improves fucoxanthin bioavailability. Fermentation directly increases beneficial compounds like GABA, short-chain peptides, and carotenoids, acting on gut-brain and neuroimmune pathways. Enhanced bioavailability ensures bioactive compounds reach the brain or act via gut routes, influencing brain function and reducing neuroinflammation/oxidative stress. WY-peptides in fermented dairy inhibit MAO-B, boosting dopamine and improving memory. GABA-rich fermented whey improves memory, brain antioxidants, anti-inflammatory cytokines, neurotransmitters, and microbiota diversity. Aglycones from fermented soy improve cognition and visual memory in postmenopausal women. Natto improved spatial learning and memory in mice by activating hippocampal TrkB/CREB signaling and increasing BDNF. Fermented soy also exhibits potent anti-inflammatory effects via genistein and NF-κB suppression. LMW fermented fucoidan improved memory/learning and upregulated BDNF/CNTF. Fucoxanthin from fermented kelp protects neurons by crossing the BBB, reducing edema, and enhancing antioxidant defenses and BDNF. Phlorotannins and alginate-oligosaccharides also contribute to neuroactivity and prebiotic effects.	Limited human *in vivo* bioavailability studies explaining specific mechanisms. Optimization of fermentation processes or microbial strains to understand the bioavailability of each bioactive compound across different food types. How individual variations, such as gut microbiota composition or digestive enzymes, affect the bioavailability and efficacy of FF components in humans is unclear. FFs contain many transformed compounds and live microbes. The synergistic or antagonistic interactions among these components, which contribute to bioavailability and cognitive effects, are not fully understood. The precise number of bioavailable compounds needed to achieve consistent cognitive benefits in humans, and its correlation with FF intake, has not yet been defined. Dose–response relationships for bioavailable compounds are lacking in the literature.
Characterization	Highlights are that FFs contain beneficial bioactive compounds beyond just the microbes themselves. Both Interventional Studies (InSs) and Observational Studies (ObSs) show a strong emphasis on milk-based fermented drinks and dairy products. Despite the dairy dominance, a significant portion of studies also included plant-based fermented foods. Some InSs specifically analyzed and quantified certain bioactive compounds. Limited details on product characteristics. For commercially available products and those produced by the food industry, quality control measures are generally presumed.	Lack of specific microbial composition for bifidobacteria. Insufficient detail on production and processing conditions. Limited nutritional composition data in InSs (plant-based) and no information in ObSs. Vague information on analytical methods, batch-to-batch variation and quality control. Lack of specific bacterial strain information in ObSs. Reproducible evaluations of key quality parameters are lacking in both InS and ObSs. Quality assessment remains inadequate in most InSs and ObSs. Sensory tests are missing for almost all of the studies.

Supplementary Tables S5, S6: Health-Related Factors and Lifestyle Associated Factors.

### 3.2 Intervention

#### 3.2.1 Type of food

In InSs, a variety of FFs were tested. This included milk fermented by *Lacticaseibacillus paracasei* strain Shirota (47), *Lactobacillus helveticus* (42), kefir (48), and skim milk powder fermented with *Lactobacillus helveticus* IDCC3801 (43). Soy-based fermented products included tempeh (45) and *Lactiplantibacillus plantarum* C29-fermented soybean powder (46). Other types of FFs involved seaweed *Laminaria japonica* and *Saccharina japonica* fermented with *Levilactobacillus brevis* BJ20 (41, 44) (Table 3).

**Table 3:** Intervention description and control description and quality.

Author, year	Type of food fermented with *Lactobacillus* spp. and *Bifidobacterium* spp.	Dosage/Frequency/Consumption/follow up	Control
Reid et al. (41)	Fermented seaweed “*Laminaria japonica*” (FSW) by *Levilactobacillus brevis* BJ20	One capsule (1.5 g of powder) daily 6 weeks	Low-quality control Sucrose pills with a lack of the same nutrient content as treatment
Hwang et al. (46)	DW2009 - a mixture of fermented soybean powder and *Lactiplantibacillus plantarum* C29 freeze-dried powder	800 mg of powder in pills, daily 12 weeks	Low-quality control No information about nutrient content of placebo cellulose capsules
Ohsawa et al. (42)	*Lactobacillus helveticus*-fermented milk -fermenting skim milk with a starter culture containing *L*. *helveticus* CM4.	One bottle (190 g per bottle) of the drink daily 8 weeks	Good quality control Similar in taste, texture, and nutrient content as treatment, lacking active microbial ingredient
Handajani et al. (45)	Tempeh A lower count of bacteria Tempeh B higher count of bacteria (*Enterobacteriaceae* and Lactic acid bacteria).	100 g of tempeh daily 6 months	Low-quality control Lack of the same nutrient content
Cannavale et al. (48)	Kefir containing *Lactobacillus lactis*, *Lacticaseibacillus rhamnosus*, *Streptococcus diacetylactis*, *L. plantarum*, *Lacticaseibacillus casei*, *Saccharomyces florentinus*, *Leuconostoc cremoris*, *Bifidobacterium longum*, *B. breve*, *B. lactis*, *Lactobacillus acidophilus, Limosilactobacillus reuteri*	236 mL of kefir daily 4 weeks	Low-quality control Lactose-free 1% low-fat milk, differ in taste and consistency from treatment
Benton et al. (47)	Milk drink fermented by *L. casei* Shirota	65 mL of drink daily 20 days	Good quality control Similar in taste, texture, and nutrient content as treatment, lacking active microbial ingredient
Chung et al. (43)	*L. helveticus*-fermented milk (LHFM) IDCC3801	500, 1,000 or 2000 mg of *L. helveticus* daily 12 weeks	Good quality control Similar in taste, texture, and nutrient content as treatment, lacking active microbial ingredient
Park et al. (44)	*Saccharina japonica* fermented by *Levilactobacillus brevis* BJ20.	1,000 mg of powder in pills daily 4 weeks	Low-quality control Lactose pills with a lack of the same nutrient content as treatment
Park et al. (56)	Yoghurt and cheese	Cut-offs for consumption: 0, 0.1–0.37, 0.38–0.74, 0.75–1.34 and 1.34 cup	Non consumers
Han et al. (62)	Fermented dairy product (yogurt, cheese, or either one of them)	The daily fermented dairy intake from low to high consumers was: 46.52 ± 27.29 g/day 137.95 ± 32.90 g/day 349.12 ± 149.49 g/day	Non-consumers
Ylilauri et al. (61)	Fermented dairy, cheese	Guided food recording of 4 days 22 year follow up Fermented dairy Intake, g/day (Quartiles): Q1 < 24 g/day Q2 24–106 g/day Q3 107–285 g/day Q4 > 285 g/day	NA, Quartiles of consumption
Kesse-Guyot et al. (53)	Yogurt, cheese	Consumption Mean (SD): yogurt: men 84.0 (75.3) g/d women 85.0 (76.3) g/d cheese: men 53.2 (33.5) g/d women 36.3 (26.4) g/d Follow up 5 years (2007–2009)	NA Low, medium and high consumption
Ni et al. (52)	Fermented; all types of yogurts and cheese	The median consumption from the lowest to the highest tertile was: Total yogurt, [g/day], median [IQR]i T1 5 (0, 13) T2 55 (51, 59) T3 127 (122, 133) g/day. Total cheese [g/day], median [IQR]j T1 10 (5, 14) T2 26 (23, 31) T3 48 (42, 59) g/day 2-year follow-up	NA, Tertiles of consumption
Muñoz-Garach et al. (58)	Fermented dairy products	Consumption categorized into quartiles: “Very low” Q1 (<220 g/day), “Low” Q2 (221–307 g/day), “Low to Moderate” Q3 (308–499 g/day) and “Moderate to High” Q4 (≥500 g/day).	Non-dairy foods
Hogervorst et al. (57)	Tempeh	Mean weekly intake: 9.5+/−6.8 times/week 65% of participants used tempeh once or more than once daily	Tofu
Hogervorst et al. (49)	Tempeh	Daily intake of tempeh (7 times a week) for 1 month	NA
Tessier et al. (50)	Regular and low-fat yogurt and regular and low-fat cheese. Fermented dairy intake was calculated as the sum of cheese and yogurt intake.	Quartiles of intake frequency: Yogurt Q1: 0.17 ± 0.25 times/d, Q2: 0.38 ± 0.36 times/d, Q3: 0.61 ± 0.39 times/d, Q4: 0.75 ± 0.48 times/d Cheese: Q1: 0.32 ± 0.27 times/d, Q2: 0.46 ± 0.27 times/d, Q3: 0.60 ± 0.35 times/d, Q4: 0.80 ± 0.53 times/d Fermented dairy: Q1: 0.49 ± 0.35 times/d, Q2: 0.84 ± 0.42 times/d, Q3: 1.21 ± 0.44 times/d, Q4: 1.55 ± 0.74 times/d	NA, Quartiles of consumption
Kim et al. (60)	Processed cheese Fresh cheese White mold cheese Blue mold cheese Other	Cheese intake: Daily 27.6% every 2nd day 23.7% 1–2 times a week 29.7% No intake 19.0%	Non-cheese intake group
Suzuki et al. (59)	Cheese: Camembert Other (processed cheese, fresh cheese, blue cheese, or other cheese.)	Cheese intake group consumed cheese at least 1–2 times per week (85% of participants)	Non-cheese intake group
de Goeij et al. (54)	Fermented dairy, total yoghurt, total cheese, Dutch cheese	Frequency and portion size - grams per day. Average daily nutrient intakes were calculated Medians with interquartile ranges (IQR): Total yoghurt 18–146 g/day Total cheese 20–47 g/day Dutch cheese 13–34 g/day Buttermilk 0–40 g/day Fermented dairy 75–235 g/day	Non consumers
Ortega et al. (55)	Yogurt and cheese	100 g per day of fermented dairy products was consumed by the participants. Follow up in 5, 9 and 13 years	NA, comparison between the food types

IQR: Interquartile range; LAB: Lactic acid bacteria; NA: Not applicable; SD: standard deviation.

The majority of ObSs were based on dairy products while only two studies (49, 57) examined soy-based food products (tempeh) (Table 3). Several studies (50, 52, 54–56, 58, 61), investigated both fermented and non-fermented dairy products, but with distinct effects on cognition. One study (54) classified dairy by fermentation status and fat content (e.g., full-fat vs. low-fat, fermented *vs*. non-fermented milk, yogurt, and cheese), and one (55) analyzed dairy across fermented, non-fermented, sugary, and fat-based categories. Some studies clustered findings by specific fermented dairy types, such as cheese (59), yogurt and cheese (61, 62) or as cheese and non-cheese (60), which were examined independently (Table 3).

#### 3.2.2 Dosage

The daily amounts of FF administered in InSs varied widely (Table 3). Regarding those in liquid form, participants were asked to consume daily 65 mL of probiotic milk (47), a bottle of 190 g of fermented milk (42) and a much higher quantity of 236 mL (8 oz) for the kefir (48). Only one study (43) was examining dose–response using pills at three different dosages (i.e., 500 mg, 1,000 mg and 2000 mg/day), though no definitive minimal threshold was established. In another, (46) fermented soybean in powder was used in a dosage of 800 mg/day. Fermented extracts of seaweed were administered in doses of 1.5 g/day (41) and 1,000 mg/day (2 × 500 mg capsules) (44). Finally, whole-food intervention of 100 g/day of two types of tempeh was implemented (45).

In ObSs, intake levels were estimated using food frequency questionnaires (FFQs) and reference pictures with standard portion sizes (53, 61). In some cases, a dietitian guided the estimation process (61). However, other studies lacked clarity in quantification methods (52, 58) or used simple assumptions, such as reporting in cup servings (56). For soy foods, the frequency of tempeh consumption was measured by the number of servings per week (49, 57). All ObSs stratified consumption amounts by tertiles, quartiles or frequency categories, which allowed for some gradient analysis. Nevertheless, none of the studies defined a specific minimum intake associated with measurable health outcomes. Due to the nature of ObSs microbial counts or microbiological composition of FF was also not reported. Instead, FF intake was assessed on the basis of product categories (yogurt, cheese, tempeh) without specifying the presence or content of live microorganisms.

#### 3.2.3 Duration of intervention

The length of intervention in InSs ranged from short-term (20 days in (47)) to long-term durations of 6 months (45). Common durations included 4 weeks (44, 48), 6 weeks (41), 8 weeks (42), and 12 weeks (43, 46), indicating a wide variation depending on the food type and study objectives. Follow up was not included in any of the InSs (Table 3).

ObSs did not implement controlled intervention durations but instead assessed the habitual or long-term dietary intake of FF using retrospective or prospective data collection. Tempeh consumption was recorded as weekly servings in the Indonesian cohort reflecting stable, self-reported long-term dietary behavior (49, 57).

Among the 13 ObSs, 5 incorporated follow-up assessments (52, 53, 55, 60, 61). Ylilauri et al. (61) reported three follow-ups over a 22-year period, with cognitive assessments performed four years after baseline. Ortega et al. (55) conducted cognitive follow-ups at 5, 9, and 13 years after baseline. Kesse-Guyot et al. (53) reported a 13-year follow-up, whereas Kim et al. (60) and Ni et al. (52) included follow-ups two years post-baseline. These repeated assessments strengthened the reliability of temporal inferences in those studies. Other studies, such as Ni et al. (52) and Tessier et al. (50), used cross-sectional or short-term prospective designs to capture dietary exposures via FFQs or dietary recalls. Suzuki et al. (59) relied on dietary recall data to assess cheese intake but did not include repeated measures or follow-up. In all cases, while intervention durations were not applicable, the dietary data aimed to reflect habitual intake patterns, allowing for the investigation of long-term associations with cognitive outcomes.

#### 3.2.4 Control conditions

Based on the assessment of the control criteria (see Methods section), three categories of control conditions were defined: ideal, high-quality and low-quality control conditions. None of the included studies met the criteria for ideal control condition (Table 3).

Three of the InSs used high-quality placebo controls that matched in taste, texture and nutrient content but did not contain live bacteria so that the effects of the food matrix and fermentation could be distinguished. Two studies used placebo beverages for the fermented milk treatment (42, 47), and two studies used placebo tablets with the same but unfermented food matrix (43). These controls, free of *L&B* or other microbes, allowed a clearer attribution of the cognitive effects to microbial fermentation. In most cases, *Lactobacillus* was the only fermenting microbial agent in the intervention, so these studies were well suited to isolate its specific effects on cognitive function.

Five InSs were classified as low-quality controls due to differences in taste, texture or nutritional content compared to the treatment. These controls could not replicate the sensory and nutritional properties of FF and limited the ability to isolate microbial and fermentation effects. In three studies, the placebo pills contained lactose, sucrose or cellulose, that do not match the nutritive status of the intervention pills (41, 44, 46). Handajani et al. (45) used low-protein cookies without soy as a placebo for tempeh, while Cannavale et al. (48) used lactose-free low-fat milk as a control for kefir, although there were clear differences in taste, texture as well as slight differences in nutritional composition. Such controls reduce the validity of conclusions about fermentation-specific effects.

None of the ObSs met the criteria for adequate controls as they were based on broad, non-specific comparisons. Controls included tofu for tempeh (57), non-dairy foods for fermented dairy products (58) or different consumption levels (49, 50, 52, 53, 55, 61). Although these controls were suitable for assessing the general effects of FF, they were not sufficient to isolate the effects of *L&B* fermentation. Three studies compared dairy consumers with non-consumers and investigated associations between total consumption of dairy products, fermented dairy products, yogurt or cheese and cognitive performance (54, 56, 62).

#### 3.2.5 Outcome

Considering the research question for this systematic review, the main outcome of the selected studies was the impact of food fermented with *L&B* on cognitive function in a healthy adult population.

Among the 8 InSs reviewed, 7 reported positive effects of FF on cognition, and one negative outcome was observed, while 10 ObSs reported positive associations, and 3 reported no (55) or adverse (52, 58) associations with at least one cognitive domain (Table 4).

**Table 4:** Cognitive outcomes and assessment tools.

Aim/objective of study Author	Method for cognitive measurement and related cognitive domain	Cognitive domain	Outcome	Positive Effect
Interventional studies				
Reid et al. (41) Considering the neuroprotective potential of fermented *Laminaria japonica* A. (FSW), as supplement that can be administered later in life to offset neurodegenerative conditions associated with aging.	K-MMSE Numerical Memory Test Raven’s Standard Progressive Matrices Flanker Test Iconic Memory Test Trail Making Test (TMT) BDNF concentrations	Global cognition Executive function (working memory, selective attention and cognitive fluidity) Processing speed Episodic memory (hippocampal-dependent) Iconic or visual sensory memory	Intervention improved global cognition, executive function, hippocampal dependent memory and iconic memory. Significant increase in BDNF serum levels	Yes
Hwang et al. (46) To assess the efficacy and safety of *Lactobacillus plantarum* C29-fermented soybean (DW2009) as a nutritional supplement for cognitive enhancement in individuals with MCI.	The verbal learning test (VLT): Auditory continuous performance test (ACPT) Digit span test (DST) BDNF concentrations	Episodic memory (hippocampal-dependent) Executive function (working memory and attention)	Intervention improvement executive function and hippocampal-dependent verbal episodic memory. Significant increase in BDNF serum levels	Yes
Ohsawa et al. (42) To determine the effects of a *Lactobacillus helveticus*-fermented milk drink containing lactononadecapeptide (NIPPLTQTPVVVPPFLQPE) on the cognitive function of healthy middle-aged adults.	Japanese version of Repeatable Battery for the Assessment of Neuropsychological Status (RBANS)	Episodic memory (hippocampal-dependent), Executive function (attention)	Intervention improved both executive function and hippocampal-dependent visual episodic memory.	Yes
Handajani et al. (45) This study aims to find the effect of consuming tempeh as a controlled intervention on cognitive function of older people, by comparing 2 types of tempeh available in the Indonesian marketplace.	Tool kit from the Consortium to Establish a Registry for Alzheimer’s Disease (CERAD) comprising: Mini Mental State Examination (MMSE), Boston Naming Test (BNT) Categorical verbal fluency test, Word list memory recall	Global cognition Episodic memory (hippocampal-dependent) Semantic memory	Both interventions improved global cognitive function, regardless of bacteria count. A significant improvement in semantic memory was only seen in the group consuming Tempeh A	Yes
Cannavale et al. (48) To determine whether consumption of a fermented dairy beverage containing probiotic microorganisms influences negative mood states, stress, and hippocampal memory performance in healthy adults	Computerized spatial reconstruction task for assessing hippocampal-dependent relational memory	Episodic memory (hippocampal-dependent)	Consumption of a fermented dairy beverage over 4 weeks is beneficial for hippocampal-dependent relational memory function.	Yes
Benton et al. (47) To evaluate the impact of consuming a probiotic-containing drink, rather than a placebo, on mood. In addition, as poor mood and poor memory are known to be related (Phelps, 2006), this aspect of cognition was also assessed.	Wechsler Memory Scale (1998). The ability to recall the capital cities of several countries Verbal fluency test National Adult Reading Test (NART)	Executive functions (long-term and working memory) Semantic memory Pre-morbid intelligence, Verbal episodic memory (hippocampal dependent)	Intervention resulted in a slightly-poorer performance on two measures of episodic memory. No difference to the memory scores after 10 days, but at 20 days placebo group had significantly better episodic memory scores	No
Chung et al. (43) This study was designed to investigate the effects of a processed skim milk powder fermented by a probiotic, *L*. *helveticus* IDCC3801 (LHFM), on cognitive functioning in healthy older adults.	Digit-span test (DST), Story recall test, Verbal-learning test (VLT) Rapid visual information-processing (RVIP) task, Stroop color-word test Brain-derived neurotrophic factor (BDNF) levels	Executive function, (attention, short term and working memory, cognitive flexibility and inhibition) Episodic memory (hippocampal-dependent)	Intervention significantly improved episodic memory	Yes
Park et al. (44) The effect of FSJ (fermented *Saccharina japonica*) on cognitive function in healthy participants and to elucidate the mechanism underlying these effects in humans.	Korean Wechsler Adult Intelligence Scale (K-WAIS) (Digit Span, Digit Symbol Coding, and Block design measures), Operation-word span task Raven’s test-based quantitative EEG test.	Executive function (working memory, attention, visual-motor coordination) Processing speed Visuospatial cognition (hippocampal-dependent)	There was no significant difference between control and intervention groups in any of the tests. The intervention treated group had significantly increased spatial memory, processing speed, and executive functions after the intervention compared to baseline.	Yes
Observational studies				
Park et al. (56) This study aimed to determine the potential relationships between the intake of dairy foods (total dairy products, milk and cheese) and cognitive function through information garnered in the National Health and Nutrition Examination Surveys (1988–94 and 1999–2002).	System (simple reaction time task (SRTT), Digit–symbol substitution test (DSST) Serial digit learning task (SDLT)). Story recall test (SRT)	Episodic memory (hippocampal-dependent) Implicit memory (procedural memory) Executive function (attention) Processing speed	Cheese consumers compared to non-consumer improved executive function and processing speed for age between 20–59 and the highest quintiles of consumption verbal episodic memory was improved for age 20–59 and 60+ Executive function and processing speed was improved in participants over 60 yrs. of age	Yes
Han et al. (62) This study examined the relationship between fermented dairy intake and cognitive function in this population.	Alzheimer’s Disease Registry Word List Learning Test (CERAD-WL), Digit Symbol Substitution Test (DSST).	Verbal episodic memory (hippocampal dependent) Executive function (attention, processing speed) Semantic memory	Yogurt consumers had higher global cognition and semantic memory scores. Cheese consumers had significantly lower Verbal episodic memory. Fermented dairy consumers showed significantly higher semantic memory and executive function, processing speed and lower episodic memory. Participants with low and medium consumption of fermented dairy show significantly higher semantic memory and executive function, as well as significantly lower episodic memory compared to non-consumers.	Yes
Ylilauri et al. (61) To investigate if fermented dairy intake is associated with dementia and cognitive performance.	Mini Mental State Exam, MMSE Trail making test A, Verbal fluency test, Selective reminding test, Russell’s adaptation of the visual reproduction test.	Global cognition Executive functions (attention, mental flexibility) Processing speed Verbal learning and memory Visual memory Semantic memory	Although none of the tests showed significant improvement in global cognition, higher cheese intake was associated with lower risk of incident of dementia, a measure of cognitive health.	Yes
Kesse-Guyot et al. (53) To examine the cross-time associations of total and specific dairy product consumption with cognitive performance in aging adults.	RI-48 test semantic fluency task phonemic fluency task forward and backward digit span tests. Delis-Kaplan trail-making test (TMT)	Episodic memory (verbal hippocampal-dependent memory) Semantic memory Executive function (working memory, attention, mental flexibility)	Positive association with executive functions (working memory) in higher cheese consumption in the minimally adjusted model. Higher yogurt and cheese consumption was significantly associated with better verbal memory performance (hippocampal-dependent memory).	Yes
Ni et al. (52) To assess the short-term longitudinal associations between milk and dairy product consumption overall and by subcategories (e.g., fat content, fermented, or nonfermented), with subsequent changes over a 2-year follow-up in cognitive performance in an older Spanish population at high cardiovascular disease risk.	Mini–Mental State Examination (MMSE), Verbal Fluency Tests (VFTs), Digit Span Tests (DSTs) of the Wechsler Adult Intelligence Scale-III (WAIS-III), Clock Drawing Test (CDT), Trail Making Tests (TMTs).	Global cognition Semantic memory Executive function (working memory, attention) Processing speed Visual–spatial function	Associations of fermented dairy (yogurt, cheese, total), with lower global cognition were significant in a crude model, but not after multivariable adjustment. Higher yogurt intake was significantly associated with greater decline in the semantic memory.	No
Muñoz-Garach et al. (58) To examine the association between milk and dairy products intake and the prevalence of cognitive decline among Spanish individuals at high cardiovascular risk.	MMSE Mini–Mental State Examination	Global cognition	Higher intake of fermented dairy products was observed in participants with a lower global cognition. The consumption of fermented dairy products was also associated with an increase in the odds of presenting dementia. Quartiles of fermented dairy product consumption showed that a higher consumption of fermented dairy products was related to an increase in the odds of worse global cognitive function.	No
Hogervorst et al. (57) To investigate the association between phytoestrogen intake and memory function in elderly men and women from urban and rural sites on Java.	The Hopkins Verbal Learning Test (HVLT)	Episodic memory (hippocampal dependent)	Tempeh was independently significantly related to better verbal episodic memory. High tempe consumption was independently related to better verbal episodic memory particularly in participants over 68 years of age.	Yes
Hogervorst et al. (49) Revisited rural Central Java where was previously found the strongest negative associations of tofu consumption with immediate recall in those over 68 years of age (57) to further investigate the association of different soy products (tofu, tempeh) with memory function	The modified Hopkins Verbal Learning Test (HVLT)	Episodic memory (hippocampal dependent)	Higher weekly tempeh consumption was associated with better verbal episodic memory. While tempe consumption had no significant associations with memory by itself, it seemed to exert protective effects only when entered in analyses with tofu consumption and could potentially offset some of its negative effects.	Yes
Tessier et al. (50). The aim of this study is to examine the association between total dairy intake and 3 cognitive domains in a large contemporary cohort of community-dwelling older Canadians. The secondary objective was to investigate associations with specific dairy types: milk, yogurt, cheese, regular-fat and low-fat, and fermented products.	15-word Rey Auditory Verbal Learning Test im mediate recall (RAVLT-I) 5-min delayed recall (RAVLT-II) Mental Alternation Test Victoria Stroop test (interference/dot) Event- and time-based prospective memory tests Controlled Oral Word Association Test Mean response time of the choice reaction time psychomotor speed	Episodic memory (hippocampal dependent) Executive function (working memory, attention, mental flexibility)	Positive cross-sectional association between total dairy product, cheese, and low-fat dairy product intake frequencies and the executive function domain, and between yogurt intake and the verbal episodic memory. Yogurt was the only dairy product significantly and independently associated with the verbal episodic memory.	Yes
Kim et al. (60). The purpose of this study was to elucidate the relationship between cheese intake and cognitive function, evaluated based on MMSE (mini-mental state examination) scores in community-dwelling older people, using cross-sectional data.	Mini–Mental State Examination (MMSE).	Global cognition	This study suggests that cheese intake is inversely associated with the risk of lower global cognitive function, even after adjusting for multiple confounding factors.	Yes
Suzuki et al. (59). This study aimed to explore the association between cheese intake/type and cognitive function, assessed using the mini-mental state examination (MMSE) in a Japanese observational cohort of community-dwelling older women.	Mini–Mental State Examination (MMSE).	Global cognition	The Camembert cheese and other cheese intake groups had an improved global cognition. In all three models, Camembert cheese intake was significantly associated with mild cognitive decline, and may prevent mild cognitive decline.	Yes
de Goeij et al. (54). The aim of this study was to investigate associations between a broad variety of dairy subclasses and dairy products with domain-specific cognitive performance in Dutch adults aged ≥65 years	MMSE Digit Span forward and backward from the Wechsler Adult Intelligence Test Trail Making Test (TMT) A&B Stroop Color-Word Test part-III Letter Fluency test Stroop Color-Word Test part-I, Symbol Digit Modalities Test (SDMT) Rey Auditory Verbal Learning Test recognition (RAVLT)	Global cognition Executive function (working memory, attention, mental flexibility) Processing speed Episodic memory (hippocampal dependent)	Higher intake levels of fermented dairy were associated with better executive functioning scores. Higher Dutch cheese intakes were associated with better executive functions.	Yes
Ortega et al. (55). Assessing the effect on cognitive function of adding dairy (total, fermented, non-fermented, full fat, low fat, and sugary) to the diet and of substituting some food groups for dairy.”	Clinical dementia rating (CDR), Cognitive Complaint Inventory, Buschke and Groeber test. Animal naming task Stroop color test Dénonimation Orale d’Images (DO40) test CERAD (Consortium to Establish a Registry for Alzheimer’s Disease)	Global cognition Episodic memory (hippocampal dependent) Executive function (working memory, attention, mental flexibility) Semantic memory Verbal memory	Addition of 100 g/d dairy (fermented or non-fermented) to the diet had no effect on cognitive function among older adults. Substituting different food groups for dairy (fermented or non-fermented) had no consistent and precise effect on cognitive function.	No effect

**Figure 2:**
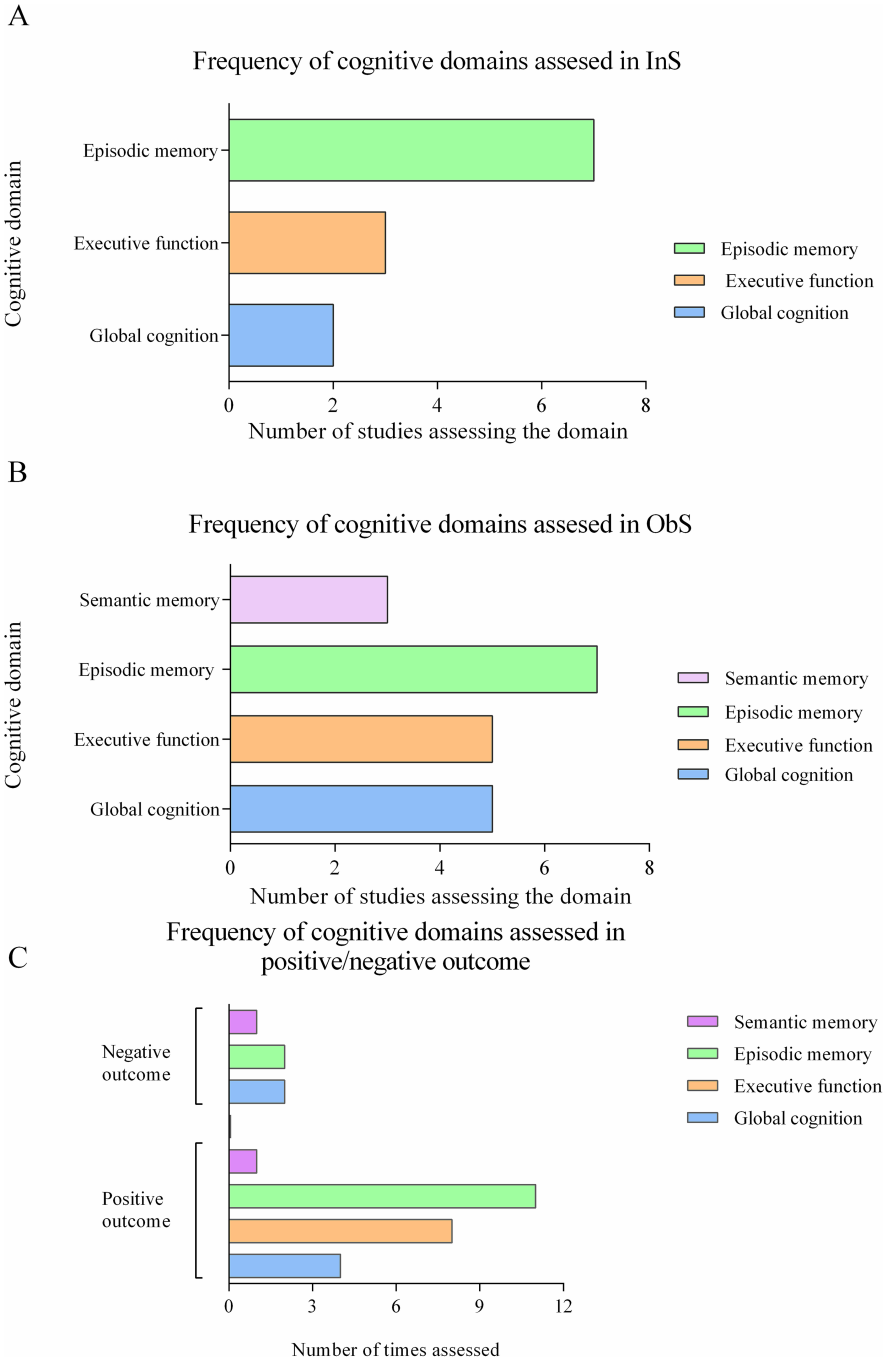
Frequency of cognitive domains used in studies. **(A)** Frequency of cognitive domains assessed in Interventional studies. **(B)** Frequency of cognitive domains assessed in Observational studies. **(C)** Frequency of cognitive domains assessed in positive and negative outcome.

Most InSs reported improvements in episodic memory, executive functions, and global cognitive function (GCF) (Table 4 and Figure 2A). Hippocampal-dependent episodic memory improved in six InSs (41–44, 46, 48), mostly assessed by verbal and visual memory tests (VLT, VLMT, HVLT, RAVLT, CERAD-WL, SRT, relational memory test, RI-48). The GCF, assessed by MMSE, improved in two studies (41, 45). Processing speed and executive functions (NMTest, TMT, ACPT, DST, DSST, verbal fluency, RVIP, Stroop, operation-word span) improved in three studies (41, 42, 46). Only Benton et al. (47) found that fermented milk slightly worsened episodic memory after 20 days.

In ObSs, consumption of cheese, yogurt and tempeh was consistently associated with improvements in episodic memory, executive function and global cognition. Most ObSs showed benefits for episodic memory; Hogervorst et al. (49, 57) found that higher tempeh intake improved verbal memory, especially in older adults. Kim et al. (60) and Suzuki et al. (59) reported an inverse association between cheese consumption and cognitive decline/dementia risk (MMSE), respectively, while Ylilauri et al. (61) found that fermented dairy did not improve memory but reduced dementia incidence over a 22-year period. Tessier et al. (50) and Kesse-Guyot et al. (53) associated yogurt and cheese with better verbal and working memory.

However, two PREDIMED studies associated higher yogurt consumption with lower GCF (MMSE) and impaired semantic memory (52) and higher consumption of fermented dairy products with lower GCF and increased risk of dementia (58). Ortega et al. (55) found no consistent cognitive benefit, suggesting neutral effects.

Cognitive outcomes varied by FF type and study design (Supplementary Table S7). Among InS, fermented dairy improved hippocampal-dependent episodic memory in three (42, 43, 48), worsened it in one (47), and improved executive memory in one case (42). Four ObS associated fermented dairy with better episodic memory (53, 56, 61, 62), four with improved executive function (53, 55, 56, 61) and two with better GCF (59, 60). One ObS found that higher cheese consumption reduced the risk of dementia (61); another found no causal relationship (54).

Plant-based FFs improved episodic memory in three InSs (41, 44, 46), executive memory in two (41, 46) and GCF in one (45). Two ObSs associated daily tempeh consumption with better verbal episodic memory (49, 57).

#### 3.2.6 Cognitive assessment

Episodic memory was the most frequently investigated domain in InSs (7 studies), followed by executive function (3 studies) and global cognition (2 studies) (Table 4 and Figure 2A). Semantic memory and visual-sensory memory were examined in two and one InS, respectively, but no effects were observed. In the ObSs, the focus was also on episodic memory (7 studies), followed by executive function and global cognition (5 studies each) (Figure 2B).

Across both study types, positive effects were reported in 11 studies for episodic memory, in 8 for executive function, in 4 for global cognition and in 1 for semantic memory (Figure 2C). Negative effects were reported in two studies each for executive function and global cognition and in one study for semantic memory.

Among cognitive tests, the MMSE was used most frequently in both InSs and ObSs, followed by the DST and VLT in InSs, and the TMT and Digit Span in ObSs. Although no single instrument can be recommended as universally optimal, the MMSE was the most commonly used, probably due to its simplicity and wide range of application. However, its limited sensitivity to subtle changes suggests that it may be suboptimal for InSs. Tests targeting episodic memory and executive function, domains most responsive to FF interventions, are recommended for future studies.

### 3.3 Quality and bias of the human studies

Only studies with high-quality control (see Methods section) were eligible for Q&B assessment. RoB was assessed using the RoB2 tool in several domains, including bias due to the randomization process, bias due to period and carryover effects, bias due to deviation from the planned interventions (effect of assignment to intervention), bias due to missing outcome data, bias in the measurement of the outcome, and bias in the selection of the reported outcome (Table 5).

**Table 5:** Quality and bias.

RoB 2 tool	Chung et al. (43)	Ohsawa et al. (42)	Benton et al. (47)
Risk of bias arising from the randomization process	Low	Low	Low
Risk of bias arising from period and carryover effects			/
Risk of bias due to deviations from the intended interventions (effect of assignment to intervention)	Low	Low	Low
Risk of bias due to deviations from the intended interventions (effect of adhering to intervention)	Low	Low	/
Risk of bias due to missing outcome data	Low	Low	Low
Risk of bias in measurement of the outcome	Low	Low	Low
Risk of bias in selection of the reported result	Low	Low	Some concerns
Overall risk of bias	Low	Low	Some concerns

Of the 21 studies included in the analysis, Q&B assessment was conducted for 3 InSs. In particular, the studies by Ohsawa et al. (42) and Chung et al. (43) were assessed as having a low RoB. In contrast, the InsS of Benton et al. (47) was rated as questionable, mainly due to problems related to the selection of reported outcomes and the randomization process.

None of the ObSs met the criteria for high-quality control and were therefore excluded from the RoB assessment.

After summarizing the results from human studies, we further investigated the mechanisms of action, bioavailability, characterisation of the FFs and their bioactive compounds and safety, according to the EFSA guidelines for the assessment of health claims.

### 3.4 Mechanism of action

While it has long been believed that the central nervous system governs cognition, recent studies have pointed to additional influencing factors, such as lifestyle choices (1). A lower risk of cognitive decline has been associated with dietary components, including B group vitamins, polyphenols, and micronutrients such as iron. Modulation of brain function through the microbiota-gut-brain axis (MGBA) also recently came to the foreground, the gut microbiota being beneficially modulated by prebiotics and probiotics. In this context, FFs are promising vehicles for dietary modulation of cognitive health, being an amalgamation of beneficial microbes as well as relevant bioactive compounds that provide both systemic health benefits as well as being specific to brain function, often through modulation of the MGBA (63). Indeed, FFs are usually rich in neurotransmitters, neuroactives and neuromodulators (64) that can stimulate the connections in the MGBA, including the immune, neuroendocrine, circulatory and enteric nervous systems. Importantly, cognitive decline has been connected to dysbiosis of the gut microbiota, which can contribute to direct inflammatory stimulation, production of pro-inflammatory metabolites and loss of immune regulation, leading to a state of chronic low-grade inflammation; FFs and related bioactive compounds and microbes have been shown to improve such a state (63, 65).

#### 3.4.1 Human evidence for mechanisms

Direct mechanistic evidence from human studies included in this systematic review is limited. Some InSs and ObSs mentioned potential mechanisms for the observed effects of FFs, such as fermented dairy (fermented milk, yogurt, kefir and cheeses), fermented soy products (tempeh) and FSW on cognition (Table 6), but the information was scarce and largely speculative.

**Table 6:** Potential bioactive compounds and MOA mentioned in the InSs, ObSs and other studies.

Study type	Food type	Bioactive compounds	Potential MoA
InSs	Fermented sea tangle (FST)	GABA; other compounds: amino acids (alanine, valine, glycine, and leucine), fermented sulfated polysaccharides (fucoidans), polyphenols (e.g., phlorotannins, carotenoids: fucoxanthin)	Crossing BBB (110), prevention decrease of pCREB expression and BDNF level that promoting neurogenesis and synaptic plasticity (Reid et al., 2018b), increased SOD (41, 44, 70, 112) and CAT (70, 112), suppressed Aβ (44), increased GPx, and GSR, reduced lipid peroxidation biomarkers (TBARS) (41, 112) and MDA (41, 70, 112) and *γ*-GT (70) and oxidative DNA damage marker 8-oxo-dG (41, 112). Reduction in angiotensin converting enzyme levels (112).
	Fermented soy products	Isoflavones and saponins	Increased BDNF level (46, 139, 140); suppression of Aβ, inhibited acetylcholinesterase activity (139), inhibition of chemically-induced NF-κB (140)) increased SOD activity, isoflavone interaction with the estrogen receptor (67, 68), gut microbiota modulation (139), Increased SOD activity (141)
	Fermented dairy products	GABA, bioactive peptides such as (NIPPLTQTPVVVPPFLQPE),	Modulation of gut microbiota such as an increase in *Lactobacillus*, *B. adolescentis* and *Phascolarctobacterium* (48). No significant change in BDNF level and whole blood viscosity regarding cardiovascular diseases (43)
ObSs	Fermented dairy	Bioactive peptides [particularly tryptophan- and cysteine-rich peptides, alpha-lactalbumin, fatty acids, vitamins (B12, D, K, K2), tyramine, minerals (e.g., calcium), lactic acid bacteria, poly-unsaturated fat in cheese, bioactive lipids (e.g., oleamide and dehydroergosterol)]	Inactivating MAO-B in the brain, raising dopamine level *via* bioactive tryptophan rich peptides (142), increase in anti-inflammatory cytokines *via* GABA, gut modulation and increase in SCFA (e.g., acetate) (105)
	Fermented soy products	Genistein and Folate	Augmentation of intestinal bioavailability and bioactivity of isoflavones, indirect effect on cerebrovascular health. Via angiotensin converting enzyme inhibition, crossing BBB, reduction in Aβ aggregation, suppressing NF-κB signaling, neural oxidative damage and inflammation in the brain (104)

Aβ: Amyloid Beta; BDNF: Brain-Derived Neurotrophic Factor; BBB: Blood–Brain Barrier; CAT: Catalase; GABA: Gamma-Aminobutyric Acid; GPx: Glutathione Peroxidase; GSR: Glutathione Reductase; *γ*-GT: Gamma-Glutamyl Transferase; MAO-B: Monoamine Oxidase B; MDA: Malondialdehyde; NF-κB: Nuclear Factor Kappa-B; SCFA: Short Chain Fatty Acids; SOD: Superoxide Dismutase; TBARS: Thiobarbituric Acid Reactive Substances; 8-oxo-dG: 8-oxo-2′-deoxyguanosine.

Fermented Dairy Products: Three InSs showed beneficial effects of fermented dairy on cognitive function. Chung et al. examined the impacts of *Lactobacillus helveticus*-fermented milk (LHFM) by investigating Brain-Derived Neurotrophic Factor (BDNF) as a possible biological mechanism through which LHFM might provide positive effects on cognitive performance. Nevertheless, no measurable GABA was detected in the product used for the intervention, indicating that the cognitive-enhancing effects could be linked to unknown compounds produced during the fermentation process by *L*. *helveticus* IDCC3801 (43). Similarly, a dairy beverage fermented by *L&B* was found to improve relational memory, with the study noting a 235% increase in *Lactobacillus* in the gut, suggesting successful colonization and beneficial impact on gut microbiota (48). Ohsawa et al. showed positive effect after treatment of lactononadecapeptide, present in milk fermented with *L*. *helveticus*, without any further information on mechanism of action and bioavailability (42).

Fermented Soy Products: Only one InS with fermented soy product gives potential MoA, by reporting the increased levels of BDNF while study on Tempeh did not mention any mechanistic/bioavailability details (45). *Lactiplantibacillus plantarum* C29-fermented soybean (DW2009) has been shown to be a safe and effective nutritional supplement for improving cognitive function in individuals with mild cognitive impairment, with effects associated with increased serum levels of BDNF (46).

Fermented Seaweed Products: Reid et al. conducted a study on fermented seaweed (FSW) that contained 54.5 ± 0.071 mg of GABA per gram of the product, with participants receiving a daily dose of 2.4 g (which equates to approximately 131 mg of GABA per day) (41). FST significantly enhanced serum BDNF levels and the antioxidant activity of GPx, GSR, and SOD, while decreasing the production of TBARS and reducing 8-oxoDG levels. Furthermore, FST also protects against the degenerative effects of aging on short-term memory and cognitive impairment associated with dementia. The bioactive constituents of FST such as GABA and fucoidan acting to provide improvements in antioxidant activity following FST supplementation may protect against progressive degeneration purportedly caused by reactive oxygen species (41). In another InS, the findings of Park et al. (44) offer emerging evidence that the potential positive impact of FSW on neurocognitive function indicated by alterations in concentration and perception capabilities is mainly based on the modulation of antioxidant activity.

*Human studies not included in our review:* Isoflavones, which are abundant in soy-based fermented products, contribute to neuromodulation through estrogen-like protective effects (66), interacting with the estrogen receptors in the brain and improving memory and cognitive function, particularly in postmenopausal women (67, 68). Moreover, a 12-week administration of 10^8^ colony-forming units (CFU)/mL *Limosilactobacillus fermentum* A2.8 isolated from tempeh, a soy-based fermented product containing LAB and *Rhizopus*, in cognitively impaired elderly subjects led to improvements in memory function, learning process, and visuospatial and verbal fluency (69). The strain *L*. *fermentum* A2.8 has been identified as carrying a glutamate decarboxylase gene, suggesting its capability to synthesize GABA, which could be the basis for its cognitive benefits. Consuming tempeh may boost cognitive functions by fostering the development of beneficial gut microbiota, which produces substances like butyrate that can increase BDNF levels. Furthermore, the intake of tempeh has also been associated with lower levels of amyloid-beta (Aβ) accumulation, which can protect neurons from damage and alleviate memory deficits (69).

Reid et al. (41) mentioned their earlier studies (70, 71) with FSW to interpret and underline the mechanism; FSW supplementation in middle-aged women stimulated the release of muscle-related growth factors, increased BDNF (71), and decreased lipid peroxidation (41). In healthy male individuals, FSW administration (1.5 g/day for 4 weeks) enhanced antioxidant defense by lowering serum levels of *γ*-glutamyltransferase and malondialdehyde (MDA), while increasing the activities of superoxide dismutase (SOD) and catalase (CAT) (70). In good agreement, Choi et al. demonstrated that GABA-enriched FSW supplementation in middle-aged women stimulates the release of muscle-related growth factors, increasing BDNF (71).

*Critical Assessment:* However, critical evaluation reveals significant limitations. The GABA doses provided by FSW (131 mg/day) are substantially lower than therapeutic doses used in clinical studies (500–750 mg daily for anxiolytic effects). Oral GABA has poor BBB penetration (<5%), questioning the biological plausibility of direct GABAergic cognitive effects at typical consumption levels (72).

#### 3.4.2 Animal and *in vitro* evidence for mechanisms

Most of the mechanistic evidence for fermented dairy, soy, and seaweed products is derived from animal studies and *in vitro* research, which might not directly translate to human physiology.

Fermented Dairy Products: Fermented dairy remains the most widely studied FF with several animal and in vitro studies elaborating on its impact on cognitive modulation. In murine models, neuroinflammation induced and associated with cognitive decline can be mitigated through the use of milk fermented with *Lactobacillus,* resulting in cognitive recovery (73). Gut inflammation and reduced intestinal barrier integrity contribute to neuroinflammation and BBB integrity reduction, and several studies have shown that FFs positively impact this phenomenon (63), with some *Lactobacillus* having the potential to preserve intestinal integrity (74). In a murine model subjected to scopolamine treatment, ethanol precipitate derived from *L*. *helveticus* IDCC 3801 LHFM significantly alleviated deficits in memory performance through modulation of amyloid precursor protein (APP) processing and presence of bioactive peptides (75).

Kefir, a dairy based FF with complex microbiome, improves performance in memory-associated tasks in murine models. Van De Wouw et al. demonstrated that kefir administration reduced immune response, increased gut microbiota capacity to produce GABA, and increased the relative abundance of beneficial *Lachnospiraceae bacterium* A2 in the gut (76, 77). More specifically, one kefir type restored stress-induced loss of colonic serotonin, while another improved fear-contextual memory with enhanced GABAergic and serotonergic signaling, indicating neurotransmitter production beyond GABA by the gut microbiota (76). Relatedly, kefiran, an exopolysaccharide (EPS) produced by *Lactobacillus kefirofaciens* in milk kefirs, has immunomodulatory properties beneficial for intestinal inflammation and barrier integrity (78).

Fermented Soy Products: Yoo and Kim demonstrated that soybean powders fermented with *Lactobacillus* spp. offered protection to mice against memory impairment induced by scopolamine, enhancing BDNF expression and reducing acetylcholinesterase activity in the hippocampus (79). Similarly, in transgenic mice, fermented soybean enhanced cognitive function and diminished the expression of Amyloid-beta (Aβ) (80). *Lactiplantibacillus plantarum* C29 increased BDNF levels and inhibited chemically induced NF-κB activation in the hippocampus, along with memory improvement in mice (80). Additionally, the alleviated memory impairment effect of *Lactiplantibacillus plantarum* C29-fermented soybean (DW2009) was also attributed to modulation of gut microbiota (81). BDNF expression might therefore be regulated by the gut microbiota changes induced by DW2009 or by neurotransmitters or its derivatives synthesized by DW2009.

Fermented Seaweed Products: The fermentation of *Saccharina japonic*a utilizing *Levilactobacillus brevis* BJ20 (FSW) has been shown to significantly augment cognitive function and memory via neuroprotective mechanisms and modulation of critical neurotrophic factors in rodent models. FSW influences its outcomes by preserving neuronal health, restoring essential brain biochemicals, and promoting neuronal growth, which ultimately enhances learning and memory functions (82). In mice, *L*. *japonica* extract fermented with *L*. *brevis* BJ20 (50 mg/kg) administered for 21 days ameliorated physical stress-induced reductions in proliferating cells and neuroblasts in the dentate gyrus, preventing decreases in BDNF and phosphorylated cAMP response element-binding protein (pCREB) expression levels (83). GABA-enriched FSW improved cognitive impairment and neuroplasticity in scopolamine- and ethanol-induced dementia model mice (83).

*Critical Assessment:* Animal research often employs doses that are 10–50 times greater per unit of body weight compared to human studies, particularly when metabolic rate is taken into account (84, 85). Rodent gut microbiomes differ fundamentally from humans in composition, diversity, and metabolic capacity (86, 87). The model of memory deficit induced by scopolamine illustrates an acute pharmacological disruption, contrasting with the gradual cognitive decline typically seen in the aging process of humans (88). For isoflavones, animal studies use concentrations of 50–200 mg/kg body weight, requiring humans to consume 2–8 kg of fermented soy daily to achieve equivalent exposure (89, 90).

#### 3.4.3 Mechanistic pathways across fermented foods by *L&B*

Overall, the potential mechanisms on how cognition is impacted across the FFs investigated in this study may be summarized across several potential pathways such as: (i) MGBA: foods fermented with *L&B* influence the gut-brain axis by modulating gut microbiota composition, enhancing the production of neuroactive compounds like GABA and SCFAs, and improving intestinal barrier function. These changes can lead to reduced systemic inflammation and improved neurotransmission, benefiting cognitive functions, (ii) Neurotransmitter modulation: Probiotic LAB strains in FFs can produce or modulate neurotransmitters. For instance, *L*. *fermentum* A2.8 from tempeh produces GABA, known for its soothing effects on the nervous system. Similarly, the intake of kefir has been associated with enhanced GABA production, which aids in boosting mood and cognitive function, (iii) anti-inflammatory effects: persistent inflammation is linked to a decline in cognitive abilities. FFs with LAB can reduce pro-inflammatory cytokines, thereby mitigating neuroinflammation. For instance, fermented soy products have been demonstrated to lower levels of TNF-*α* and Interleukin 6 (IL-6), aiding in neuroprotection, (iv) antioxidant properties: oxidative stress damages neurons and hinders cognitive performance. FFs enhance antioxidant defenses by increasing the activity of enzymes like SOD and CAT and reducing oxidative markers like MDA. FSW has demonstrated significant antioxidant effects in both animal and human studies, and (v) neurotrophic factor enhancement: BDNF is crucial for neurogenesis and synaptic plasticity. FFs such as yogurt, kefir, and fermented soy whey have been found to elevate BDNF levels, enhancing cognitive functions like memory and learning.

Our research yielded significant evidence that supports the likely beneficial impacts of *Lactobacillus* spp. in FFs on cognitive health, primarily through modulation of the MGBA highlighting that FFs act as a vehicle for dietary modulation of cognitive health by influencing the MGBA. This encompasses advantageous modifications to the gut microbiome, resulting in overall health improvements and enhanced cognitive function. FFs can directly or indirectly increase the production of neuroactive compounds. The intake of kefir has been associated with increased GABAergic and serotonergic signaling, which plays a role in better memory and mood in murine models (48). *Limosilactobacillus fermentum* A2.8, isolated from tempeh, produces GABA, potentially explaining its cognitive effects (69).

*Critical Evaluation:* Among the various proposed mechanisms, MGBA modulation emerges as the most biologically credible pathway, although it still necessitates consistent intake over prolonged durations (91). The well-documented two-way communication between the gut microbiome and the brain suggests that changes in the microbiome can occur within weeks after dietary adjustments (92). Nevertheless, the majority of studies employ undefined mixed bacterial cultures instead of specific therapeutic strains, and the therapeutic threshold for probiotic effects generally requires 10^9^ colony-forming units daily, a target that may not be reliably met through the consumption of FFs alone (93, 94).

*Dose–Response Relevance:* Clinical studies examining GABA-mediated mechanisms have administered doses between 200 mg and 1,500 mg daily to elicit positive outcomes (95, 96), while commercial products typically suggest a daily intake of 600–750 mg (72). This amount is considerably greater than the GABA concentrations generally present in natural and FFs, which are unlikely to reach therapeutic levels (97). Considering that the average intake of these foods provides only 50–150 mg of GABA each day and that oral GABA demonstrates limited ability to cross the BBB (<5%) and is quickly metabolized, it appears that the direct cognitive impact of GABA is biologically implausible at standard consumption levels (72).

*Individual* Var*iability:* Genetic variations in cytochrome P450 enzymes influence the metabolism of numerous bioactive compounds present in FFs (98, 99). The foundational composition of the gut microbiome shows significant variability among individuals, impacting the generation of bioactive metabolites and their responses to dietary changes (100, 101). The metabolism of isoflavones in humans differs according to the composition of the gut microbiome, with 30–50% of individuals lacking the ability to produce equol, which is the most bioactive isoflavone metabolite (102, 103).

The mechanistic insight into the impact of FFs on cognitive function indicates that the majority of the suggested pathways exhibit limited biological relevance at doses applicable to humans. The MGBA stands out as the most promising mechanism, yet it necessitates consistent intake over prolonged durations and is influenced by substantial individual variability. For healthcare professionals, moderate effects may be attainable through the consistent, long-term use of FFs, but individual reactions will vary greatly, and immediate cognitive enhancement is improbable at standard consumption levels. Future investigations should focus on human pharmacokinetic research involving critical bioactive compounds and mechanistic biomarker studies that directly assess the proposed pathways in human subjects.

#### 3.4.4 Bioavailability

The shift from consuming FFs to experiencing cognitive advantages is largely reliant on bioavailability—the degree to which bioactive compounds can be absorbed, distributed, and delivered to their intended sites to produce biological effects. Understanding this complex process uncovers both the potential and the constraints of FFs as means to enhance cognitive performance.

Fermentation represents a remarkable biological change that fundamentally alters the bioavailability attributes of food matrices. Upon examining the FFs included in this systematic review—dairy, soy, and seaweed products—a consistent trend can be observed: fermentation converts native compounds into forms that are more bioactive or easily absorbed, frequently *via* the hydrolysis of glycosides or the decomposition of large polysaccharides into smaller, more manageable molecules (104). This change is more than just a chemical reaction; it represents a biological improvement that enhances the ability of these substances to influence human physiology.

The process is perhaps most elegantly illustrated in fermented soy products, where lactic-acid or fungal fermentation transforms isoflavone glycosides like genistin and daidzin into their respective aglycones—genistein, daidzein, and glycitein (104). This transformation greatly enhances intestinal absorption, since aglycones possess much higher bioavailability compared to their glycosylated forms. Likewise, the fermentation of dairy proteins liberates bioactive peptides that would otherwise remain confined within larger protein structures, while bacterial or enzymatic treatment of seaweed polysaccharides such as fucoidan and carrageenan diminishes their molecular weight, substantially improving their absorption potential.

These fermentation-related alterations boost the concentrations of bioactive compounds such as GABA, short peptides, and carotenoids including fucoxanthin, which can enter the bloodstream. Notably, these compounds possess the capability to either cross the blood–brain barrier directly or affect brain function via vagal and gut pathways (105, 106). The resultant impacts on gut-brain and neuroimmune pathways encompass enhancements in gut barrier integrity, alterations in microbiota composition and neurotransmitter synthesis, decreases in neuroinflammation and oxidative stress, and the upregulation of neurotrophic signaling pathways, particularly BDNF. Together, these mechanisms create a biological foundation for enhancing memory, focus, and neuroprotection.

Fermented dairy products like yogurt, cheese, and fermented milks represent one of the most extensively studied categories of FFs, and their bioavailability attributes provide valuable understanding of how fermentation can enhance compounds that are advantageous for cognitive performance. During the fermentation of dairy, short “lactopeptides” derived from casein and whey proteins are produced through the activity of bacterial proteolytic enzymes.

Numerous peptides in this category exhibit the extraordinary capacity to traverse the gut-brain axis and influence neurotransmission directly, creating a straightforward link between dietary intake and neurological impact.

The cognitive advantages derived from bioactive peptides produced during the fermentation of dairy have been demonstrated in one of the InSs in our systematic review (42) in Table 4, providing human evidence for this bioavailability pathway. Alongside peptides, various fermented dairy products are enhanced with GABA via the microbial decarboxylation of glutamate (107), leading to items with enhanced neuroactive properties.

The ability of oral GABA sourced from fermented dairy products to be absorbed, and its effectiveness have been verified in animal studies, where GABA notably enhanced both novel object recognition and working memory (108). In models involving aging or brain injuries, whey that is rich in GABA bolstered brain antioxidant defenses and stimulated the production of anti-inflammatory cytokines, while simultaneously reducing markers of oxidative stress and inflammation, thereby reinstating neurotransmitter levels in the brain and fostering cellular autophagy (105). These advantages were associated with the enhancement of gut microbiota diversity and a rise in the synthesis of acetate, a valuable short-chain fatty acid that supports brain health (105).

Nevertheless, applying these encouraging findings from animal studies to human biology is still a challenging endeavor. Although the research illustrates a definitive bioavailability of GABA and peptides derived from fermented dairy products, the levels reached in typical human consumption situations might be significantly lower than those utilized in animal experiments, prompting inquiries regarding the clinical significance of these mechanisms at usual consumption levels.

The narrative of bioavailability related to fermented soy products serves as a prominent example of how fermentation can reveal therapeutic benefits. Soybeans inherently possess elevated levels of isoflavones, yet these substances are generally attached to sugar molecules in their glycoside forms, which considerably restricts their bioavailability and biological efficacy. The intervention of LAB and *bifidobacteria* fundamentally alters this picture by modifying these isoflavones into more bioactive and bioavailable aglycone forms (104).

This change carries significant consequences for cognitive well-being. Genistein and daidzein, the main aglycone forms generated during fermentation, are phytoestrogens that can traverse the blood–brain barrier and stimulate neuroprotective mechanisms. Animal models demonstrate that these soy isoflavones can reduce amyloid-beta aggregation, oxidative stress, and inflammation in brain tissue (104), while estrogen-like signaling in the hippocampus enhances synaptic plasticity—a fundamental mechanism underlying learning and memory.

The importance of these increased bioavailability levels is corroborated by research conducted on humans. A meta-analysis encompassing 10 intervention trials revealed modest yet noteworthy advancements in overall cognitive function and visual memory among postmenopausal women who were consuming soy supplements (109). This observation implies that the increased bioavailability attained through fermentation could lead to substantial cognitive advantages, especially in groups experiencing reduced endogenous estrogen levels. The cognitive benefits of isoflavones were observed not exclusively for postmenopausal females, but also for premenopausal females as well as males (138).

In addition to isoflavones, fermented soybean products enhance cognitive health via various bioavailability mechanisms. The fermentation process decomposes soy proteins into bioactive peptides, including lunasin and other smaller peptides that may help in reducing blood pressure (104). These peptides could indirectly promote cognitive health through cardiovascular pathways. Moreover, fermented soy has been shown to mitigate neuroinflammation by inhibiting nuclear factor kappa-B (NF-κB) signaling (104) and lessening neural oxidative damage, thereby offering multiple avenues through which increased bioavailability might lead to cognitive improvements.

These mechanisms that enhance bioavailability may explain the epidemiological findings that associate high soy and fermented soy diets with a deceleration in cognitive aging (104, 109), indicating that the improvements in bioavailability resulting from fermentation hold significant implications for maintaining cognitive health.

FSW constitutes a distinct category of FFs that presents both unique bioavailability challenges and opportunities. The fermented soy tested in our systematic review (41) includes fucoidan, a sulfated polysaccharide that, in its raw state, is too large to efficiently enter systemic circulation. Nevertheless, fermentation cleavages these large molecules into smaller fragments that can be absorbed and might exert biological effects.

Park et al. and Zhang et al. illustrated that fucoidan-rich substances derived from FSW are capable of crossing the BBB and providing neuroprotective benefits (82, 110). This signifies a remarkable achievement in bioavailability, considering that the BBB typically inhibits large polysaccharides from passing through. The fermentation process appears to yield molecular fragments that are perfectly sized for both intestinal absorption and BBB penetration.

The bioavailability profile of FSW extends beyond just fucoidan, encompassing additional marine bioactives. Fucoxanthin, a notable carotenoid present in brown seaweed, demonstrates enhanced bioavailability following fermentation. This compound has the ability to cross the BBB and offer direct neuroprotective effects, as evidenced by animal studies where fucoxanthin administration led to reductions in brain edema, lesion volume, and dendritic loss while also stabilizing the BBB by preserving tight junction proteins (111). The fermentation process can improve the bioavailability of fucoxanthin by breaking down algal cell walls and freeing bound carotenoids.

FSW also releases polyphenols like phlorotannins, which have demonstrated the ability to reverse memory deficits induced by scopolamine through the ERK-CREB-BDNF signaling pathways in animal studies (112). Moreover, fermentation boosts the concentration of alginate-oligosaccharides, which act as prebiotics to aid advantageous gut bacteria, thus possibly fostering cognitive health through MGBA pathways.

The bioavailability of FFs is closely linked to the specific microbial strains engaged in the fermentation process; however, this crucial aspect is frequently inadequately articulated in most studies. Among the fermentation strains discussed in our review, FST was created using *Levilactobacillus brevis* BJ20, whereas the soybean powder DW2009 was a combination of *Lactiplantibacillus plantarum* C29 fermented soybean powder and freeze-dried bacterial powder (46). Fermented dairy products incorporated various strains, including *Lactobacillus helveticus* IDCC3801 44, *L*. *helveticus* CM4 43, and *Lacticaseibacillus paracasei* strain Shirota (47).

It is essential to recognize that while lactobacilli are prevalent in the fermented products present in InSs, bifidobacteria are significantly deficient. The commercially available kefir used in one study (48) is the sole product fermented with *Bifidobacterium* species, featuring a diverse blend of 12 live probiotic cultures that includes *Bifidobacterium longum*, *B*. *breve,* and *B*. *animalis* subsp. *lactis*. This absence of representation raises concerns, since different bacterial strains can produce distinct bioactive compounds and influence bioavailability in ways that are unique to each strain.

The implications of strain selection on bioavailability become clear when analyzing the production of specific compounds. Reid et al. monitored GABA production using HPLC, validating an average GABA concentration of 54.5 ± 0.071 mg/g in their fermented seaweed product (41). In a similar manner, Ohsawa et al. measured the lactononadecapeptide content in their fermented milk (42). However, many studies failed to provide comprehensive profiles of bioactive compounds, which limits our understanding of the specific contributions of different strains to bioavailability.

The gathered evidence suggests that consistently consuming fermented dairy, soy, or seaweed products can enhance the diet with elements that demonstrate superior bioactivity and heightened bioavailability, contributing to the maintenance of cognitive function and the stabilization of mood (Table 6).

*Critical Evaluation:* The bioavailability of FF compounds exhibits considerable individual variation that is seldom recognized in existing research. Variations in genetic polymorphisms of drug-metabolizing enzymes, discrepancies in gut microbiome composition, alterations in intestinal permeability, and individual differences in gastric pH and transit duration can significantly influence the bioavailability of FF compounds.

The distinctive composition of a person’s gut microbiome significantly influences its capacity to transform daidzein into equol, a metabolite with greater biological activity, particularly when it comes to isoflavones sourced from fermented soy products. About 30–50% of people do not possess the necessary gut bacteria for equol synthesis, which may account for the diverse cognitive responses seen in studies involving soy interventions. Likewise, variations in GABA metabolism and the permeability of the BBB could elucidate the inconsistent cognitive impacts noted with GABA-rich fermented dairy products.

The present landscape of bioavailability research in FFs reveals significant gaps that need to be addressed in order to formulate evidence-based recommendations for cognitive well-being. Upcoming research should focus on thorough pharmacokinetic studies that monitor bioactive compounds from ingestion through their absorption, distribution, metabolism, and eventual elimination. These investigations ought to incorporate assessments of plasma and cerebrospinal fluid concentrations of essential bioactive compounds to determine whether cognitively significant levels are attained in human consumers.

The bioavailability of compounds associated with cognitive function derived from FFs is a complex interplay that includes molecular alterations caused by fermentation, specific microbial activities tied to certain strains, individual physiological variations, and factors influencing product quality. Although fermentation evidently improves the bioavailability of numerous bioactive compounds when compared to their unfermented counterparts, there are still significant gaps in our knowledge regarding dose–response relationships, variations among individuals, and how bioavailability correlates into clinically significant cognitive outcomes.

The results suggest that FFs possess the capability to deliver bioactive compounds to targeted tissues, including the brain, through multiple pathways, which involve both the direct crossing of the blood–brain barrier and indirect mechanisms related to the gut-brain axis. Nevertheless, the levels attained through standard consumption habits may be significantly lower than those observed in animal studies that exhibit cognitive advantages, prompting critical inquiries regarding the clinical significance of the suggested mechanisms.

Future investigations need to embrace a more stringent methodology for evaluating bioavailability, integrating thorough analytical characterization, factors of individual variability, and clinically significant outcome measures. It is only through these methodologies that can be formulated as evidence-based guidelines for FF consumption that enhance both bioavailability and cognitive advantages, while recognizing the complex individual factors that affect therapeutic results.

### 3.5 Characterization of the fermented foods and their bioactive compounds

Foods fermented by *L&B* are recognized not only for their microbial content, but also for increased bioavailability of inherent bioactive components available in the matrix or produced during fermentation, including certain amino acids and their metabolites such as tryptophan metabolites, neurotransmitters (e.g., acetylcholine and GABA), vitamins (e.g., vitamin B12, vitamin B9 or vitamin K2), SCFAs, bioactive peptides, as well as polyphenols, isoflavones and phytosterols in plant-based FF. Considering the importance of microbes and bioactive compounds for the efficacy of the FF, a meticulous description on the production and processing conditions is of utmost importance, as the amount of live bacteria and the bioavailability of bioactive compounds can vary significantly, depending on the final product characteristics.

Possibly due to long tradition on fermented dairy in most countries, short fermentation time and commercial availability, milk-based fermented drinks were overrepresented in the eligible InSs (42, 43, 47, 48): commercially available kefir (48), fermented milk drink containing *Lacticaseibacillus paracasei* strain Shirota mixed with water, sugar, skimmed milk powder and flavoring, provided by Yakult (47). *Lactobacillus helveticus* CM4-fermented milk containing lactononadecapeptide, along with stabilizer, sweetener, and flavors (42), and a milk fermented with *Lactobacillus helveticus* IDCC3801, precipitated and dried, provided in a tablet (43).

However, 4 InSs included in this review also focused on plant-based FF, specifically fermented soybean (45, 46). In the InS (45) Indonesia, commercially available Tempeh was used as test products, while in the InS of Hwang et al. (46), Korea, a mixture of fermented soybean powder and *Lactiplantibacillus plantarum* C29 freeze-dried powder (DW2009) delivered in capsules, was applied. In the InS of Park et al. (44), *Levilactobacillus brevis* BJ20 FSW extract was provided as capsules, while in the study of Reid et al. (2018) (41) soft capsules including FSW, lactose, cellulose, HPC, SiO_2_, and magnesium stearate were administered as test product. Similarly of the 13 included ObSs, 11 primarily focused on fermented dairy products, encompassing various cheeses (regular-, high- and low-fat cheese, cottage cheese, Dutch cheese, processed cheese, fresh cheese, white mold cheese, blue mold cheese, other cheese) and yogurt (regular and low-fat), sometimes investigated as groups, sometimes individually (52–55, 58, 59, 61, 62). However, two of the included ObSs also assessed the influence of soy-based FF, e.g., tofu and tempeh (49, 57), on the participants’ cognitive parameters. In Table 7 an overview of all the fermented products included in this review is given, listing some of the defined characteristics.

**Table 7:** Product characterization.

Author, year	Type of food fermented with *Lactobacillus* spp. and *Bifidobacterium* spp.	Calories	Carbohydrates	Protein	Fat	Additional information	Live bacteria	Proposed bioactive compound	Dosage/Frequency/Consumption/follow up	Control
*Interventional studies*										
Reid et al. (41)	Fermented seaweed *Laminaria japonica* (FSW) by *Levilactobacillus brevis* BJ20	No info	No info	No info	No info	No info	No info	Amino acids, including alanine, valine, glycine, and leucine, sulfated polysaccharides such as fucoidans and polyphenols, GABA (54.5 ± 0.071 mg/g)	One capsule (1.5 g of powder) daily 6 weeks	Low-quality control Sucrose pills with a lack of the same nutrient content as treatment
Hwang et al. (46)	DW2009 - a mixture of fermented soybean powder and *Lactiplantibacillus plantarum* C29 freeze-dried powder	No info	No info	No info	defatted soybean (6%)	Final product contained 62.5% *L. plantarum* C29-fermented soybean powder and 37.5% freeze dried *L. plantarum* C29 powder.	1.25 × 1,010 CFU/g or more	Isoflavones, saponins	800 mg of powder in pills, daily 12 weeks	Low-quality control No information about nutrient content of placebo cellulose capsules
Ohsawa et al. (42)	*Lactobacillus helveticus*-fermented milk -fermenting skim milk with a starter culture containing *L. helveticus* CM4.	40,900 cal	7.1 g	3 g	0 g	179.1 g. moisture, 0.8 g ash	Inoculated with 3% starter culture	Lactononadecapeptide 2.4 mg (0.0027% (w/w))	One bottle (190 g per bottle) of the drink daily 8 weeks	Good quality control Similar in taste, texture, and nutrient content as treatment, but lacked active microbial ingredient
Handajani et al. (45)	Tempeh A (lower bacterial count) Tempeh B (higher bacterial count) (*Enterobacteriaceae* and LAB).	No info	No info	No info	No info	No info	Tempeh A: 1 × 10^8^ CFU/mL Tempeh B: 1 × 10^10^ CFU/mL	Isoflavone, soy protein, Enterobacteriaceae and LAB	100 g of tempeh daily 6 months	Low-quality control Lack of the same nutrient content
Cannavale et al. (48)	Kefir containing *Lactobacillus lactis*, *Lacticaseibacillus rhamnosus*, *Streptococcus diacetylactis*, *L. plantarum*, *Lacticaseibacillus casei*, *Saccharomyces florentinus*, *Leuconostoc cremoris*, *Bifidobacterium longum*, *B. breve*, *B. lactis*, *Lactobacillus acidophilus*, *Limosilactobacillus reuteri*	110 cal	12 g	11 g	2 g (1%)	Lactose-free	25–30 × 10^9^ CFU	Acetylcholine, GABA, Lactobacilli	236 ml of kefir daily 4 weeks	Low-quality control Lactose-free 1% low-fat milk, differ in taste and consistency from treatment
Benton et al. (47)	Milk drink fermented by *L. casei* Shirota	50,000 cal	12 g	0.8 g	0.1 g	No info	6.5 × 10^9^ CFU (1 × 10^8^ CFU/ml)	*L. casei* Shirota	65 ml of drink daily 20 days	Good quality control Similar in taste, texture, and nutrient content as treatment, but lacked active microbial ingredient
Chung et al. (43)	*L. helveticus*-fermented milk (LHFM) IDCC3801	No info	No info	4.5% (w/w)	No info	Lactose (83.0%, w/w), lactic acid (3.33%, w/w), citric acid (0.76%, w/w) succinic acid (0.26%, w/w), total inorganic matter (3.6%, w/w)	No info	Unknown bioactive compound as GABA production could be not confirmed	500, 1,000 or 2,000 mg of *L. helveticus* daily 12 weeks	Good quality control Similar in taste, texture, and nutrient content as treatment, but lacked active microbial ingredient
Park et al. (44)	*Saccharina japonica* fermented by *Levilactobacillus brevis* BJ20.	No info	No info	No info	No info	*“Saccharina japonica* extract mainly consists of sugar protein, amino acids, minerals, polyphenols, and dietary fiber”	No info	*Saccharina japonica, Levilactobacillus brevis BJ20,* Fucoidan, GABA	1,000 mg of powder in pills daily 4 weeks	Low-quality control Lactose pills with a lack of the same nutrient content as treatment
*Observational studies*										
Park et al. (56)	Yogurt and cheese	No info	No info	No info	No info	No info	No info	No info	Cut-offs for consumption: 0, 0·1–0·37, 0·38–0·74, 0·75–1·34 and 1·34 cup equivalents.	Non consumers
Han et al. (62)	Fermented dairy products (yogurt, cheese, or either one of them)	No info	No info	No info	No info	No info	No info	No info	The daily fermented dairy intake from low to high consumers was: 46.52 ± 27.29 g/day 137.95 ± 32.90 g/day 349.12 ± 149.49 g/day	Non-consumers
Ylilauri et al. (61)	Fermented dairy, cheese	No info	No info	No info	No info	No info	No info	No info	Guided food recording of 4 days 22 year follow up Fermented dairy Intake, g/day (Quartiles): Q1 < 24 g/day Q2 24–106 g/day Q3 107–285 g/day Q4 > 285 g/day	NA, Quartiles of consumption
Kesse-Guyot et al. (53)	Yogurt, cheese	No info	No info	No info	No info	No info	No info	No info	Consumption Mean (SD): yogurt: men 84.0 (75.3) g/d women 85.0 (76.3) g/d cheese: men 53.2 (33.5) g/d women 36.3 (26.4) g/d Follow up 5 years (2007–2009)	NA Low, medium and high consumption
Ni et al. (52)	Fermented dairy, all types of yogurts and cheese	No info	No info	No info	No info	No info	No info	No info	The median consumption from the lowest to the highest tertile was: Total yogurt, [g/day], median [IQR]i T1 5 (0, 13) T2 55 (51, 59) T3 127 (122, 133) g/day. Total cheese [g/day], median [IQR]j T1 10 (5, 14) T2 26 (23, 31) T3 48 (42, 59) g/day 2-year follow-up	NA, Tertiles of consumption
Muñoz-Garach et al. (58)	Fermented dairy products	No info	No info	No info	No info	No info	No info	No info	Consumption categorized into quartiles: “Very low” Q1 (<220 g/day), “Low” Q2 (221-307g/day), “Low to Moderate” Q3 (308–499 g/day) and “Moderate to High” Q4 (≥500 g/day).	Nondairy foods
Hogervorst et al. (57)	Tempe(h)	No info	No info	No info	No info	No info	No info	No info	Mean weekly intake: 9.5+/−6.8 times/week 65% of participants used tempeh once or more than once daily	Tofu
Hogervorst et al. (49)	Tempeh	No info	No info	No info	No info	No info	No info	No info	Daily intake of tempeh (7 times a week) for 1 month	NA
Tessier et al. (50)	Fermented dairy, all types of yogurts and cheese	No info	No info	No info	No info	No info	No info	No info	Quartiles of intake frequency: Yogurt: Q1: 0.17 ± 0.25 times/d, Q2: 0.38 ± 0.36 times/d, Q3: 0.61 ± 0.39 times/d, Q4: 0.75 ± 0.48 times/d Cheese: Q1: 0.32 ± 0.27 times/d, Q2: 0.46 ± 0.27 times/d, Q3: 0.60 ± 0.35 times/d, Q4: 0.80 ± 0.53 times/d Fermented dairy: Q1: 0.49 ± 0.35 times/d, Q2: 0.84 ± 0.42 times/d, Q3: 1.21 ± 0.44 times/d, Q4: 1.55 ± 0.74 times/d	NA, Quartiles of consumption
Kim et al. (63)		No info	No info	No info	No info	No info	No info	No info	Cheese intake: Daily 27.6% every 2^nd^ day 23.7% 1–2 times a week 29.7% No intake 19.0%	Non-cheese intake group
Suzuki et al. (59)		No info	No info	No info	No info	No info	No info	No info	Cheese intake group consumed cheese at least 1–2 times per week (85% of participants)	Non-cheese intake group
de Goeij et al. (54)		No info	No info	No info	No info	No info	No info	No info	Frequency and portion size - grams per day. Average daily nutrient intakes were calculated Medians with IQR: Total yogurt 18–146 g/day Total cheese 20–47 g/day Dutch cheese 13–34 g/day Buttermilk 0–40 g/day Fermented dairy 75–235 g/day	Non consumers
Ortega et al. (55)		No info	No info	No info	No info	No info	No info	No info	100 g per day of fermented dairy products was consumed by the participants. Follow up in 5, 9 and 13 years	NA, comparison between the food types

The only InS, which included a product fermented with *bifidobacteria*, was the study by Cannavale et al. (48) employing commercial kefir as a test product. Due to a lack of specific information on the microbial composition of products included in the ObSs, the consumption of *Bifidobacterium*-fermented products in those studies cannot be estimated. However, for raw milk cheese a certain probability is given that *bifidobacteria* is present (113).

#### 3.5.1 Raw material and processing

Since the included InSs and ObSs on fermented dairy were conducted in countries where milk production predominantly relies on cows (USA, Canada, Finland, Netherland, Switzerland, Spain, France, UK, Japan, Korea and China) and none of the studies specified otherwise, the raw material for these fermented dairy products is assumed to be primarily cow’s milk. However, regional differences of cow’s milk characteristics, country- and brand-specific recipes for cheese and yogurt production, and the use of diverse starter cultures result in a wide range of product variations. Differences in milk characteristics relate to the breed, feed, season, temperature and lactation status (114, 115) as well as the processing parameters (e.g., raw, thermised, pasteurized or UHT (Ultra-High Temperature)) (116, 117).

In France and Switzerland, a wide variety of raw milk cheeses exists, delivering a higher microbial load and a more diverse microbiota. In contrast, cheeses made with milk that have been thermised or pasteurized exhibit a lower bacterial diversity, while fresh cheeses with shorter fermentation times possess lower bacterial and metabolic abundances. Traditional cheese making techniques, such as back slopping for artisanal cheese production, as well as different ripening time also contribute to a very broad diversity of microflora. Furthermore, processed cheeses are manufactured by adding emulsifiers, vegetable oil, salt, sugar, food colorings and non-fermented milk components, leading to a distinctively different end product.

Furthermore, despite the definition by the Codex Alimentarius standard for fermented milks (118), yogurt can vary greatly and be fat-free, low-fat or full-fat, Greek-style, with addition of milk or whey protein, sugar, fruits, coffee, chocolate, nuts or other ingredients. The application of different starter cultures can also lead to variations in acidity, texture and amino acid composition. Moreover, yogurts with different adjunct cultures can vary greatly in abundance of bioactive metabolites (119).

Of the 4 studies (InSs and ObSs) on soy-based FF (45, 46, 49, 57), 3 utilized commercial tempeh and tofu available in the Indonesian market (46, 49, 57). However, market available tempeh varies in properties and bacterial count, influenced by raw materials, inoculum and production methods (45).

Tempeh fermentation generally occurs in two main phases: an initial LAB acidification of soybeans, creating optimal conditions for *Rhizopus* spp. which dominate the second phase. A crucial step is soybean soaking, however soaking time and the duration of both fermentation phases can vary widely (120, 121). Consequently, in the InS of Handajani et al. (45), two different tempeh products were compared to investigate the parameters linked to the beneficial effects of this FF. Hwang et al. (46) used fermented soybean powder, administered as capsules, as a test product.

Similarly, in the two InSs involving FSW as intervention (41, 44), preparation as capsules was employed. According to an earlier study (82) the FSW was prepared as follows: *S*. *japonica* was added to water at a ratio of 1:15 (w/v), 3% yeast extract and 1% glucose were added. After autoclaving at 121 °C for 30 min, the fermented *S*. *japonica* (FSW) was filtered, and *Levilactobacillus brevis* BJ20 (accession No. Korean Collection for Type Culture [KCTC] 11377BP) culture broth was mixed with the filtered sample (5% (v/v)) and incubated at 37 °C.

#### 3.5.2 Nutritional composition

The nutritional composition, when specified, varied highly among the fermented milk drinks applied in the InSs. Daily caloric servings ranged from 110–50,000 calories (42, 47, 48). Protein content varied between 0.8–11 grams per day, while carbohydrate content was specified at 7–12 grams per day (42, 47, 48). Fat content was consistently low, at 0–2 grams per day in these fermented milks (42, 47, 48); as shown in Table 7.

In contrast to the InSs on dairy-based FF, none of the InSs on plant-based FF defined the general nutritional composition of the fermented test products included in the studies (41, 44–46).

Due to the nature of ObSs, specific information on the nutritional value of included products is unavailable, as these vary greatly depending on raw material, starter cultures, individual processing and country- and brand-specific manufacturing processes. Nevertheless, the Codex Alimentarius defines some framework parameters, such as the minimum protein content of 2.7% for yogurt (118) or a fat dry matter content of ≥45% and ≤60% for full fat cheese (122). Standards also exist for specific cheese types (mozzarella (123), Emmental (124)), soy protein products (125), and tempeh (126).

#### 3.5.3 Microbial composition

While nutrient composition data was largely missing in the InSs, most of them did provide information on the strains used in fermentation, often including accession numbers.

Seaweed products (41, 44) for example were fermented with *Levilactobacillus brevis* BJ20 (Accession No. KCTC 11377BP). The soybean powder DW2009 (46) is a mixture of *Lactiplantibacillus plantarum* C29 fermented soybean powder and *Lactiplantibacillus plantarum* C29 freeze-dried powder. While milk used in one InS (43) was fermented with *Lactobacillus helveticus* IDCC3801; Ohsawa et al. (42), used a starter culture containing *L*. *helveticus* CM4 for fermentation of the milk containing lactononadecapeptide (NIPPLTQTPVVVPPFLQPE). Benton et al. (47) used fermented milk drink containing *Lacticaseibacillus paracasei* strain Shirota (Yakult, Japan) mixed with water, sugar, skimmed milk powder and flavoring.

While lactobacilli are omnipresent in test products of the eligible InSs, the commercial kefir in the InS of Cannavale et al. (48) is the only product fermented with *Bifidobacterium*. This commercial kefir strain consortium included 12 live probiotic cultures (*Lactobacillus lactis*, *Lacticaseibacillus rhamnosus*, *Streptococcus diacetylactis*, *Lactiplantibacillus plantarum*, *Lacticaseibacillus casei*, *Saccharomyces florentinus, Leuconostoc mesenteroides* subsp. *cremoris, Bifidobacterium longum*, *Bifidobacterium breve*, *Lactobacillus acidophilus*, *Bifidobacterium animalis subsp*. *lactis*, and *Limosilactobacillus reuteri*). Thus, fermented products containing bifidobacteria are underrepresented in the InSs reviewed. Furthermore, the information for the InS of Handajani et al. (45) is rather vague, as it provided overall contents of lactobacilli and *Enterobacteriaceae* for different commercial tempeh products, without specific strain composition.

Although lactobacilli always play a role in fermented dairy or soy products (cheese, yogurt or tempeh), information on the specific bacterial strains contained in ObSs products examined is generally unavailable. The same applies to the overall microbial content of the products investigated in ObSs, despite recent efforts to bridge this gap by considering country-specific differences (127–129). However, although a very important feature, information on the overall microbial content in the final test products is also rather rare and only given for four InSs (45–48), ranging from 6.5 × 10^9^–1.25 × 10^10^ CFU per serving. None of the studies reported variation during storage time.

On the contrary, Reid et al. (41), did not report a specific cell count but controlled directly for GABA production via High-Performance Liquid Chromatography (HPLC). The mean content of GABA was 54.5 ± 0.071 mg/g in the FSW. Ohsawa et al. (42) indicated inoculation with 3% starter culture containing *L*. *helveticus* CM4 and controlled the final fermented milk (including stabilizer, sweetener and flavors) for lactononadecapeptide content per bottle (190 g). In contrast, InS by Chung et al. (43) reported neither total microbial content nor potential bioactive components, although main compounds like lactose (83.0%, w/w), lactic acid (3.33%, w/w), citric acid (0.76%, w/w) and succinic acid (0.26%, w/w), as well as protein content (4.5%, w/w) and total inorganic substances (3.6%, w/w), were determined. Park et al. (44) described the test product as “500 mg capsules of standardized *Lactobacillus* FSJ (Marine Bio, Busan, Korea),” but no information was given on total microbial content or GABA content, despite their previous study with the same product (70).

The microbial composition observed in the final products exhibited considerable variation across different studies, with values ranging from 6.5 × 10^9^ to 1.25 × 10^10^ colony-forming units per serving among the four studies that reported this data (45–48). This variation carries significant implications for bioavailability, as the concentration of live microorganisms can influence both the production of bioactive compounds and the modification of gut microbiota composition.

#### 3.5.4 Batch-to-batch variability

Since the food industry was involved in Benton et al. (47), batch-to-batch variations in food composition were most likely controlled, though this is not explicitly described in the study. Similarly, Cannavale et al. (48) and Handajani et al. (45) used commercial kefir and tempeh products, assuming control over batch variations. However, no information is provided, and lack of brand names or exact product details prevents tracing back applicable standards.

In contrast, Hwang et al. (46) stated that quality, including shelf-life was guaranteed via “several validated analytical methods” for the mixture of fermented soybean powder, providing no further information about quality parameters, methods or batches controlled. Also, batch variation control is implied for the InSs of Reid et al. (41) and Park et al. (44), controlling for GABA content in the FSW test products, without any further information given.

Batch-to-batch control is self-evident for commercial products reported in ObSs, irrespective of the lack of explicit information.

#### 3.5.5 Analytical methods

All InSs on fermented milk products (42, 47, 48) provided nutritional compositions, implying analyses were performed, but failed to detail the analytical methods used. Only Ohsawa et al. (42) specified using Liquid Chromatography–Mass Spectrometry (LC–MS) multiple reaction monitoring (MRM) for quantifying lactononadecapeptide. Conversely, Chung et al. (43), clearly outlined analytical methods used - HPLC for lactose, lactic, citric and succinic acids and inductively coupled plasma optical emission spectrometry (ICP-OES) for total inorganic matter - while providing less extensive nutritional data. For all other InSs on fermented milk (42, 47, 48) we can only assume that Codex Alimentarius recommendations for analytical methods were followed (130).

The InS by Handajani et al. (45), analyzed the microbial content of the two commercial tempeh products, using plate count agar (PCA) for total microbial content, eosin methylene blue agar (EMB) for total coliforms and Man-Rogosa-Sharpe (MRS) agar for total LAB. Nutritional parameters were not analyzed.

In the InS of Hwang et al. (46), the product quality and shelf-life of the fermented soybean powder was guaranteed through “validated analytical methods,” but methods and parameters analyzed were not specified. However, in an earlier study (79), the HPLC quantification of various isoflavones and saponins (genistin, genistein, daidzin, daidzein, soyasaponin I and soyasapogenol B) was described for this test product.

Reid et al. (41) confirmed the GABA content of their encapsulated FSW product to be 54.5 ± 0.071 mg/g using HPLC, without determining the overall microbial content of the final soft capsules. Also, Park et al. (44) mentioned an increase in GABA during fermentation of FSW but did not specify further parameters to ensure standardization of the final test capsules.

Since various commercial products were used in the ObSs, we can assume their nutrient and microbial content were characterized using Codex Alimentarius (130) recommended methods, even though this information is not explicitly provided.

#### 3.5.6 Quality system

The involvement of the food industry and the use of commercial products (e.g., kefir, tempeh) in studies (45, 47, 48), highlights the awareness of the importance of robust quality systems for ensuring the reliability of InSs with FF. Yakult, which provided the test product in the study of Benton et al. (47) adhered to a comprehensive set of quality and safety system standards, depending on the date and location of the plant, as outlined in the Yakult Group Basic Quality Policy (131, 132). The food quality and safety systems promoted are Hazard Analysis and Critical Control Points (HACCP) (133), different international standards, focusing on quality management (ISO 9001), food safety (ISO 22000) and occupational health and safety (ISO 45001), Food Safety System Certification 22,000 (FSSC 22000) (134), Good Manufacturing Practice (GMP) (135), food quality management systems based on Islamic law (Halal) and Safe Quality Food (SQF) (131, 132).

However, Cannavale et al. (48) and Handajani et al. (45), did not specify the details for the commercial test products used, so that the traceability of the quality system standards applied is not given. Nevertheless, Cannavale et al. (48) mentions that the information on the microbes that are supposed to be present in the commercial kefir product is only moderately accurate.

Across several studies on fermented products, the level of detail regarding quality assurance varied significantly. For example, Ohsawa et al. (42) provided nutritional data but lacked details on overall quality verification, only assessing the functional component lactononadecapeptide. Similarly, Chung et al. (43) outlined analytical methods but omitted quality inspection. In contrast, Hwang et al. (46) stated product quality and shelf-life were guaranteed via “validated analytical methods,” though these methods and the specific parameters analyzed were not defined.

While Reid et al. (41) ensured that the FSW had 40–60 mg/g GABA, a sort of quality check for effectiveness, no such information was provided for Park et al. (44).

ObSs relies on commercial products, implying various quality systems like GMP or various international standards (ISO, see list above), depending on the region and product.

Interestingly, despite the potential impact of texture and sensory properties on a product’s effect, only Benton et al. (47) described a sensory evaluation for their fermented milk, assessing sweetness/sourness, wateriness/creaminess, pleasantness/unpleasantness and flavor intensity.

## 4 Discussion

### 4.1 Relationship between consumption of the fermented food investigated and functional effect

Almost all InSs reported positive effects of foods fermented with *L&B* on cognitive performance (41–46, 48). These findings were observed across multiple memory domains in self-reported healthy male and female participants who were primarily aged 60 to early 70s, a well-representative population given that age-related memory loss typically begins around the age of 50. Cognitive improvements were also observed in studies that included younger adults (≥18 years) (44, 48, 56) and in studies focusing only on individuals with MCI (45, 46).

However, lifestyle factors such as alcohol consumption, BMI/obesity, use of foods other than the FF investigated, and medication intake were reported inconsistently in the InSs, which limits the interpretation of the results. Benton et al. (47) was the only study to report a negative effect: participants who consumed a milk drink fermented by a *Lacticaseibacillus paracasei* strain Shirota recalled stories slightly worse after 20 days, while no difference was found after 10 days. Importantly, some of the 124 study participants were taking medication for diabetes mellitus, hypothyroidism or hypertension, although the exact number was not disclosed — an omission that could significantly affect the interpretation of the results. Despite the largest sample size, this study also had the shortest intervention period (20 days). Interestingly, a post-hoc analysis revealed that participants with poorer baseline memory were more likely to benefit from the treatment, while those with better memory showed improvement from the placebo, but none of them with significance.

Both men and women were included in the ObSs, with the average age of the participants being between 65 and 73 years old. Self-reported medication use, and BMI were more frequently taken into account, often as covariates. Most ObSs associated consumption of *L&B*-FFs with better cognitive performance, with exceptions in two PREDIMED-Plus cohort studies, which reported lower global cognition in individuals who consumed larger amounts of yoghurt and other fermented dairy products (52, 58). These studies were limited to individuals with metabolic syndrome, suggesting that health status may significantly modify the effects of FF on cognition and warrant careful consideration. Similarly, Ortega et al. (55) found no consistent cognitive benefit of increased consumption of fermented dairy products.

The studies by Hogervorst (49, 57) applied the “window of opportunity” hypothesis, which assumes a critical period in life when interventions are most likely to provide cognitive benefits, the conceptual framework lacking in other ObSs. They emphasized the impact of age on the cognitive effects of soy-based FF in older Indonesians. In their 2008 study, high consumption of fermented tempeh was associated with improved memory, especially with a significant effect in individuals aged ≥68 years. Conversely, the consumption of tofu (non-fermented soy product) was negatively associated with memory in this age group. A follow-up further showed that the positive effect of tempeh on memory only occurred with simultaneous consumption of tofu, possibly attenuating the negative effects of tofu. These results suggest that both age and certain FF influence cognitive outcomes, emphasizing the need for age-specific dietary strategies.

Controls varied significantly in their ability to evaluate FFs’ microbial and fermentation effects distinctly from broader nutritional effects. Dairy-based FF interventions typically used more rigorous placebo designs than plant-based interventions. Greater standardization and improved control practices in future studies are necessary to enhance clarity and reliability of results concerning FF effects.

### 4.2 Substantiation of a causal relationship between the consumption of fermented food and the functional effect

Specificity of effect refers to the clear and consistent association between the consumption of a particular food or constituent and a clear, measurable cognitive benefit. Several InSs have shown that foods fermented with *Lactobacillus* spp. led to improvements in different domains of cognition, especially in hippocampal-dependent memory, followed by executive memory and general cognition, demonstrating a causal relationship. ObSs further support the specificity, and all these results suggest that the cognitive effects are not general or random, but specific to certain FFs, strains, or product types, supporting the requirement for specificity in establishing a causal link as per EFSA guidance. Unfortunately, due to the (a) absence of ideal control for food fermented by *L&B*, (b) absence of InSs with *Bifidobacterium* spp. as solely intervention, and (c) a very low number of InSs with good Q&B, we cannot say that there is a clear and consistent causal relationship between the consumption of a food fermented by *L&B* and a cognitive benefit as EFSA guidance indicates.

#### 4.2.1 Dose–response and minimal effective dose

Most InSs provided information on the bacterial strains and the amount of FFs or powder used as intervention. However, only two studies investigated the dose–response relationship between the consumption of FFs and cognitive function. Chung et al. (43) used a multiple-dose design and administered *Lactobacillus helveticus* fermented milk at three different doses (500, 1,000, and 2,000 mg/day). The most pronounced cognitive benefits were observed at 1,000 mg/day, indicating a possible non-linear dose–response relationship. Handajani et al. (45) indirectly investigated the microbial dose–response relationship by comparing two types of tempeh with identical amounts (100 g/day) but different microbial loads without providing exact microbial levels; the product with the lower microbial content produced greater cognitive benefits, suggesting microbial composition as a dose-dependent factor. All other InSs used fixed-dose interventions, which preclude an assessment of dose–response. But without data on the bacterial levels, the effective minimum dose remains undetermined.

The ObSs did not report microbial levels, composition, or minimum effective intake, although they provided valuable insights into the consumption levels required to achieve effects compared to habitual dietary patterns. A potential dose–response relationship between FF intake and cognitive function was explored by categorizing participants based on intake frequency or quantity, typically into tertiles, quartiles, or quintiles, and comparing the effect between different quantities/frequencies. Similarly, Hogervorst et al. (49, 57) reported that higher consumption of tempeh was positively associated with memory performance, particularly in older adults, suggesting a dose-dependent benefit. De Goeij et al. (54) found that increasing tertiles of consumption of fermented dairy products were associated with improved cognitive performance, with each 30 g/day increase in Dutch cheese consumption associated with a significant reduction in the likelihood of poor cognitive performance. However, not all observational findings were consistent: Han et al. (62) found that consumers of fermented dairy products with low to medium frequency of consumption had better cognitive outcomes (verbal fluency, executive function) than consumers with high frequency of consumption, suggesting a potentially inverted U-shaped dose–response curve. Ni et al. (52) found that higher yogurt consumption was associated with deterioration in verbal fluency, and Muñoz-Garach et al. (58) observed a non-significant increase in the risk of cognitive impairment with higher consumption of fermented dairy products. Some large cohort studies [e.g., (61)] reported dose-dependent cognitive benefits of cheese. But because of the absence of information on microbial level, no definite result regarding the minimal effective dose can be concluded.

Dose–response investigations, along with comprehensive assessments of bioavailability, are essential for identifying the minimum effective doses and optimal consumption methods. These studies should consider individual variability factors, including genetic polymorphisms that affect metabolism, the inherent composition of gut microbiomes, and physiological aspects that influence absorption.

#### 4.2.2 Magnitude of the effect and its physiological relevance

The magnitude of cognitive effects refers to the extent of improvements observed, while physiological relevance refers to whether these changes translate into meaningful daily functioning or long-term health benefits. Across studies, reported cognitive benefits ranged from modest to moderate, with stronger evidence in a subset of InS that targeted areas such as episodic memory and executive functions (see Supplementary Text 1). The improvements reported were statistically significant, and when accompanied by physiological markers (e.g., increased BDNF), they suggest potential real-world benefits, including cognitive maintenance in old age or reduced risk of dementia. Effect sizes varied depending on the InSs, but the interpretation is limited by the small number of participants in most of the InSs, and lack of ethnicity data. Some reported within-group improvements from baseline to endpoint, suggesting a small effect magnitude, while others showed significant differences between groups post-intervention, suggesting a stronger effect. In most ObSs, effect size was not quantified, and results were not related to clinical thresholds, limiting the ability to assess the strength of results to different populations and FFs. Therefore, while the current evidence indicates a potentially appropriate magnitude and physiological relevance of the effect, it remains insufficient to draw firm conclusions regarding the magnitude or practical relevance in EFSA terms. There is a clear need for well-designed longitudinal studies with standardized microbial profiles and cognitive tools assessing episodic memory and executive function.

Fermented dairy-based foods (e.g., yoghurt, cheese) have been studied most frequently and have often been associated with moderate to mild benefits in both InSs and ObSs. Soy-based products (tempeh) were also promising, especially in studies with clear intervention protocols and follow-up. In contrast, other FFs (seaweed-based) showed domain-specific effects but lacked consistency and long-term data.

#### 4.2.3 Duration range and physiological effect

InSs provide the primary evidence, with several studies reporting improvements in hippocampal-dependent memory and executive functions following the consumption of fermented dairy, soy or seaweed products. The duration of the intervention ranged from 4 to 12 weeks, which is consistent with current evidence that probiotic and FF intake can influence cognition via gut-brain axis mechanisms within a few weeks (136, 137), and only one study lasted 6 months (45) (Supplementary Text 2). Fermented dairy products generally improved memory and attention after 4–8 weeks, while fermented soy and seaweed showed benefits within 6–12 weeks. The effects were more pronounced in older adults and people with MCI, especially with interventions lasting 6 weeks or longer. The results were largely consistent across studies from Japan, Korea and Indonesia. In contrast, a 20-day intervention showed mild cognitive impairment, suggesting that the duration may be too short to achieve positive effects (47). While most studies used a duration that was likely sufficient for physiological effects, none of the InSs conducted follow-up, so the sustainability of cognitive benefits presents a significant gap in the evidence.

ObSs assessed FF intake over longer periods, ranging from several months to over two decades. These studies offer valuable insight into long-term associations, although they could not establish direct causality or distinguish whether the observed cognitive differences were the result of only FF consumption or other intertwined confounding factors. Some studies suggested potential cognitive benefits with even short-term habitual intake, but others reported inconsistent or negligible effects despite prolonged exposure. As a result, although observational data provide supportive context, they are less conclusive regarding the specific duration needed for FFs to influence cognitive outcomes.

#### 4.2.4 Regional patterns in cognitive effects of fermented foods

In Indonesia, two linked studies showed that tempeh is consistently associated with improved episodic memory in older people. Fermented dairy products were frequently associated with better cognitive performance in older adults in the USA, Canada, and the Netherlands, e.g., processing speed, executive function, and verbal fluency. However, the results were not uniformly positive: large studies in Spain and Switzerland reported neutral or even negative associations, possibly due to limited cognitive testing or health-related confounders. Yoghurt consumption showed mixed results: Some studies reported an improvement in episodic memory and global cognition, while other studies found a deterioration in verbal fluency, indicating a possible dose–response relationship or cultural differences in diet. Cheese consumption was most consistently associated with cognitive benefits in six studies from Asia, Europe, and North America. These included improved episodic memory, executive function, and reduced risk of dementia in both older and younger adults, suggesting consistency across cultures and ages.

To summarize, hippocampal-dependent memory performance and executive functions are most reliably improved, with tempeh and cheese showing the strongest and most consistent effects across populations and research groups. We must emphasize that, although the study sites may allow some inference about the likely ethnicity of participants, this cannot replace direct reporting and limits our ability to assess whether the observed effects might differ by ethnic group or cultural dietary behavior. While there are geographical differences, the overall pattern suggests a degree of reproducibility that supports the cognitive benefits of FF consumption. The effects of FFs on cognitive function are partially consistent, particularly for foods such as fermented soy, cheese, and seaweed, and in cognitive domains such as episodic memory, executive function, and global cognition. These domains were most frequently improved in both InSs and ObSs, regardless of study setting or design, strengthening the evidence base.

### 4.3 Summarizing the relationship between the consumption of fermented food and its functional effects

Overall, the current body of evidence suggests a potentially positive effect of FF consumption on cognitive function, especially dairy products such as yogurt, cheese and fermented milk.

InSs frequently reported improvements in cognitive outcomes, particularly in episodic memory and executive functions. However, the lack of appropriate control, follow-up periods, small sample sizes, and the absence of a dose–response assessment including the minimum effective dose, weaken the validity of the results and makes it impossible to determine whether the observed cognitive benefits are transient or persistent, undermining the mechanistic insight, clinical relevance and rendering claims of long-term effects scientifically unreliable. In addition, mainly *Lactobacillus* spp. was used in the treatments; only one study examined *Bifidobacterium* spp., but in a mixture of other 12 bacterial strains without specifying the number of microorganisms (e.g., CFU), limiting the ability to attribute the observed effects to specific strains or dosages.

Larger and more diverse populations and a longer follow-up period support the association between habitual FF consumption and preserved cognition in ObS. These studies more often accounted for confounding factors such as lifestyle and BMI/obesity and provided data on frequency or amount of consumption. However, the results are limited by methodological issues, including the use of food frequency questionnaires, lack of quantification of the microbiome, and limited cognitive depth, with five studies using only one cognitive test.

Although there is ample evidence that consumption of foods containing *L&B* improves cognitive ability, the lack of appropriate controls leading to poor quality assurance presents a major limitation of these studies. Additionally, ethnicity, pregnancy, obesity, and eating disorders were largely unreported, and no sex-specific analyses were conducted in mixed populations, although sex distribution was reported in most studies.

#### 4.3.1 Summary of gaps and evidence across studies

This systematic review presents extensive evidence for the positive effects of FFs on cognitive health, particularly those containing *Lactobacillus* spp. (Table 8). InSs generally provided precise evaluations of improvement in episodic memory, followed by executive functions and general cognition, using well-defined clinical cognitive measures. Dairy-based interventions were more frequent, and showed robust cognitive benefits compared to plant-based interventions, primarily due to methodological rigor and standardized cognitive testing approaches. Overall, seven studies showed positive outcomes (41–46, 48), and one study showed a negative outcome on cognition (47).

ObSs provided broad associations between FF consumption and cognitive outcomes, demonstrating population-level trends. Ten studies showed positive outcomes (49, 50, 53–57, 59–62), and two studies showed negative outcomes (52, 58). For one study, reviewers reported that there was no effect, but the intervention had both positive and negative effects on the outcome, depending on the cognitive domain assessed (55).

Overall, there was significant heterogeneity in outcome selection and methodology between FF groups (dairy vs. plant-based) and between study designs (InS vs. ObS). In-depth cognitive assessment tools focusing on episodic memory, executive function and general cognition, would enhance interpretability and comparability in future studies.

Effects observed in these studies are largely mediated by modulation of the MGBA, influencing immune, neuroendocrine circulatory, and enteric nervous system. FFs induce favorable changes in the gut microbiota, increase the synthesis of neuroactive compounds (e.g., GABA, serotonin), reduce low-grade inflammation, improve antioxidant defenses and increase BDNF levels, thus modulating brain function, neurogenesis and synaptic plasticity.

Improved bioavailability in FFs also influenced the observed effect: soy fermentation converts isoflavone glycosides into aglycones; dairy fermentation releases bioactive lactopeptides and increases GABA levels; seaweed fermentation breaks down large fucoidans and improves fucoxanthin uptake. These changes help bioactive compounds reach target tissues, attenuate neuroinflammation and oxidative stress, and support brain function. Nevertheless, further investigation is essential to thoroughly comprehend the specific mechanisms involved, including the contributions of tempeh’s isoflavones and soy protein on the brain (69). Dairy-based FFs have been extensively studied, with the microbial strains often specified in the interventions and some analyses confirming the bioactive compound content.

Despite promising results, major gaps remain (Table 8). Most mechanistic evidence comes from animal studies; evidence in human studies is scarce because of limitations and difficulties of postmortem studies. The role of specific *L&B* strains in cognitive enhancement is unclear, with *Bifidobacterium*-fermented products being underrepresented. The complexity of FFs, rich in transformed compounds and microbes, makes it difficult to decipher synergistic or antagonistic effects on bioavailability and cognition.

There is insufficient evidence on which fermentation processes or microbial strains best enhance bioavailability in different foods. Individual differences (e.g., gut microbiota, genetics) influencing FF efficacy have not yet been sufficiently researched. Additionally, dose–response relationships are undefined, leaving unclear how much of a bioactive compound is needed for cognitive benefits.

In terms of characterization of FF, reproducible quality assessments are rare. While some information is often provided, it lacks depth. Analytical methods are mentioned, but usually without comprehensive quality testing. Commercial products are assumed to comply with industry standards (GMP, ISO), but traceability and details are often lacking, making independent quality testing difficult.

Some studies report the use of “validated analytical methods” to ensure product quality and shelf-life, but often lack data on nutritive, bioactive, or microbial parameters, making reproducibility difficult. While data on microbial strains (sometimes with accession numbers) is occasionally provided, the nutrient composition is often not. Sensory properties are rarely assessed. Only one study has provided a complete sensory profile for fermented milk.

Despite efforts to characterize test products, the current characterization of FFs is not sufficiently detailed for a robust quality assessment. This limitation also applies to commercial products, which often lack detailed traceability, analytical methods and sensory profiles. Addressing these gaps is critical to improve scientific rigor and reproducibility in FF research.

Current global regulations for FF often lack consistency, primarily focusing on food safety and varying significantly based on geographical, political, and cultural influences. New frameworks are needed to prioritize transparency and quality, reflecting a collaborative effort between science and policy to integrate evidence from food, diet, and health into actionable public health policies.

### 4.4 Safety

The safety and tolerability of FFs were generally well-regarded across several studies. In the InS by Hwang et al. (46), the safety and tolerability of the *Lactiplantibacillus plantarum* C29-fermented soybean supplement (DW2009) was closely monitored through regular participant visits, and no serious adverse effects reported. Similarly, Ohsawa et al. (42) observed no adverse effects such as dizziness, neurological, gastrointestinal, or skin issues in either the test or placebo groups consuming *Lactobacillus helveticus*-fermented milk.

Moreover, Benton et al. (47) noted that fermented milk is generally well tolerated, even by individuals with lactose intolerance who can consume yogurt. However, they advised that those with severe lactose intolerance should consult a healthcare provider before consumption. For the InSs on FSW (41, 44), no adverse effects were reported. These InSs suggested GABA and fucoidan as bioactive compounds but also highlighted the need for further research to avoid potential fucoidan toxicity.

Notably, the only adverse effects reported across all reviewed studies were linked to non-fermented dairy and milk consumption. Ylilauri et al. observed poorer performance in a verbal fluency test in their ObSs, particularly among APOE-ε4 carriers (one of the common genes, which plays a crucial role in transporting fats and cholesterol in the body, particularly in the brain) (61). This finding suggests a potential cognitive risk for this specific population from non-fermented dairy, while no adverse effects from FFs were noted, but further clinical studies should assess this issue.

## 5 Conclusion

### 5.1 Summary of evidence

Refined research question that was defined in the Study Protocol and concept paper (51) in the form of a health benefit in PIO terms (population, intervention, outcome) on the basis of the scientific evidence reviewed by the project is as follows:

The consumption of foods fermented with *Lactobacillus* spp. and/or *Bifidobacterium* spp. may have a potentially beneficial effect on cognitive performance in healthy adults, including individuals with MCI, but the current evidence is neither convincing nor sufficient to establish a clear causal relationship.

By evaluating the totality of the evidence for the health benefits of the foods fermented with *Lactobacillus* spp. and/or *Bifidobacterium* spp. in a qualitative manner following EFSA wordings, we conclude that the evidence presented is:

(b) “NEITHER CONVINCING NOR SUFFICIENT.”

The summary of the evidence is estimated in a qualitative manner based on the identification of the evidence and gaps for the health benefits of the FFs of interest identified in each of the sections in the Results and Discussion. Although we use the EFSA terminology to evaluate the evidence, our review is not associated with a health claim dossier that was evaluated by the EFSA.

## Data Availability

The raw data supporting the conclusions of this article will be made available by the authors, without undue reservation.

## Footnotes

1
https://osf.io/z6grw
