# Supplementary material for: The effects of Lactobacillus and/or Bifidobacterium in fermented foods on cognitive health: a systematic review
Source: Front Nutr. 2025 Dec 3;12:1682419. doi: 10.3389/fnut.2025.1682419 (PMC12708534; doi:10.3389/fnut.2025.1682419)
Supplement: Supplementary file 2 [file Data_Sheet_2.pdf]

## Supplementary material

**Supplementary Table S1.** Search strategy for bibliographic search of published human studies related to the health benefits and risks of fermented foods.

| | Query |
|---|---|
| No. | **Database: PubMed** |
| #1 | "Fermented Foods"[Mesh] OR "Fermentation"[Mesh] OR ("Food"[Mesh] AND ferment*[tiab]) OR ((ferment*[tiab] OR cultur*[tiab] OR leaven*[tiab]) AND (food*[tiab] OR drink*[tiab] OR beverage*[tiab])) OR "Fermented product"[tiab:~6] OR "Fermented products"[tiab:~6] OR "cultured product"[tiab:~6] OR "cultured products"[tiab:~6] OR "product fermentation"[tiab:~6] OR "products fermentation"[tiab:~6] OR "starter culture*"[tiab] OR ((ferment*[tiab] OR culture*[tiab] OR sour*[tiab]) AND (milk[tiab] OR dairy[tiab])) OR buttermilk[tiab] OR sour cream*[tiab] OR cheese*[tiab] OR yoghurt[tiab] OR yogurt[tiab] OR "yoghourt"[tiab] OR "yakult"[tiab] OR "quark"[tiab] OR "kefir"[tiab] OR "lassi"[tiab] OR "kumis"[tiab] OR "koumiss"[tiab] OR "kajmak"[tiab] OR "airag"[tiab] OR "ayran"[tiab] OR "calpis"[tiab] OR "borhani"[tiab] OR "chal"[tiab] OR "doogh"[tiab] OR kvass[tiab] OR skyr[tiab] OR amasi[tiab] OR bouza[tiab] OR butter*[tiab] OR chal[tiab] OR filmjolk[tiab] OR kishk[tiab] OR labne*[tiab] OR ((Ferment*[tiab] OR cultur*[tiab] OR cured[tiab]) AND (meat*[tiab] OR fish*[tiab] OR seafood*[tiab] OR shellfish[tiab] OR sausage*[tiab])) OR "salami"[tiab] OR "pepperoni"[tiab] OR peperoni[tiab] OR "chorizo"[tiab] OR "cervelat"[tiab] OR "mettwurst"[tiab] OR "summer sausage"[tiab] OR "sucuk"[tiab] OR "dried meat"[tiab] OR "dried sausage"[tiab] OR "dry sausage"[tiab] OR "fish sauce"[tiab] OR "shrimp paste"[tiab] OR "shrimp sauce"[tiab] OR "oyster sauce"[tiab] OR "prosciutto"[tiab] OR "pancetta"[tiab] OR "saucisson"[tiab] OR sucuk[tiab] OR ((Ferment*[tiab] OR cultur*[tiab]) AND (fruit*[tiab] OR vegetable*[tiab] OR coconut*[tiab] OR almond*[tiab] OR hazelnut*[tiab] OR nut[tiab] OR cucumber*[tiab] OR lemon*[tiab] OR citrus[tiab] OR cabbage*[tiab] OR cauliflower*[tiab] OR pepper*[tiab] OR carrot*[tiab] OR olive*[tiab] OR onion*[tiab] OR sago[tiab])) OR "sauerkraut"[tiab] OR "table olive"[tiab] OR pickle*[tiab] OR "kimchi"[tiab] OR "paocai"[tiab] OR torshi[tiab] OR ((Ferment*[tiab] OR cultur*[tiab]) AND (tea[tiab] OR teas[tiab] OR juice*[tiab])) OR "beer"[tiab] OR "wine"[tiab] OR "cider*"[tiab] OR shochu[tiab] OR "kombucha*"[tiab] OR "pulque"[tiab] OR puer[tiab] OR pu'er[tiab] OR pu-er[tiab] OR pu-erh[tiab] OR "pu erh"[tiab] OR "fuzhuan"[tiab] OR "dark tea*"[tiab] OR "yellow tea*"[tiab] OR coffee[tiab] OR shalgam[tiab] OR hardaliye[tiab] OR ((Ferment*[tiab] OR cultur*[tiab]) AND (soy[tiab] OR soya[tiab] OR bean*[tiab] OR pea[tiab] OR peas[tiab] OR lentil*[tiab] OR chickpea*[tiab] OR legume*[tiab] OR pulse*[tiab] OR (poi[tiab])) OR "soy sauce"[tiab] OR "soya sauce"[tiab] OR "soybean paste"[tiab] OR "miso"[tiab] OR "tempeh"[tiab] OR tempe[tiab] OR "natto"[tiab] OR "doenjang"[tiab] OR "doubanjiang"[tiab] OR douchi[tiab] OR "gochujang"[tiab] OR cheonggukjang[tiab] OR tsukemono[tiab] OR garri[tiab] OR ((Ferment*[tiab] OR cultur*[tiab] OR leaven*[tiab]) AND (cereal*[tiab] OR grain*[tiab] OR wheat*[tiab] OR oat[tiab] OR oats[tiab] OR rice*[tiab] OR millet*[tiab] OR sorghum*[tiab] OR maize*[tiab] OR rye[tiab] OR barley*[tiab] OR chia[tiab] OR oilseed*[tiab] OR teff[tiab])) OR "bread"[tiab] OR "sourdough"[tiab] OR "crispbread"[tiab] OR "boza"[tiab] OR "ogi"[tiab] OR dosa[tiab] OR "tarhana"[tiab] OR "buckwheat"[tiab] OR "spelt"[tiab] OR "einkorn"[tiab] OR "quinoa"[tiab] OR "amaranth"[tiab] OR "tef"[tiab] OR "bushera"[tiab] OR chica[tiab] OR chicha[tiab] OR choujiu[tiab] OR injera[tiab] OR mahewu[tiab] OR ogiri[tiab] OR pozol[tiab] OR ugba[tiab]) OR ((Ferment*[tiab] OR cultur*[tiab]) AND ("condiment*"[tiab] OR relish*[tiab] OR horseradish[tiab] OR "dressing*"[tiab] OR "seasoning*"[tiab] OR "sauce*"[tiab] OR cocoa*[tiab] OR tuber[tiab] OR "acetic acid"[tiab])) OR "chocolate*"[tiab] OR "vinegar*"[tiab] OR "tabasco"[tiab] OR "sriracha"[tiab] OR "Worcestershire"[tiab] OR "Worcester"[tiab] |
| #2 | "Diet"[Mesh] OR "Life Style"[Mesh] OR "Eating"[Mesh] OR "Feeding Behavior"[Mesh] OR ((food[tiab] OR macronutrient*[tiab] OR eating[tiab]) AND (intake*[tiab] OR habit*[tiab] OR behavior*[tiab] OR pattern*[tiab])) OR diet*[tiab] OR intake[tiab] OR ingestion[tiab] OR suppl*[tiab] OR consumption[tiab] OR meal*[tiab] OR nutrient*[tiab] OR nutrit*[tiab] |

| | |
|---|---|
| #3 | “cognition” [Majr] OR cogniti* [tiab] OR cogniti* [MeSH] OR memory [tiab] OR memory [MeSH] OR brain [tiab] OR brain [MeSH] OR attention [tiab] OR attention [MeSH] OR nerv* OR nerv* OR learning [tiab] OR learning [MeSH] OR intelligence [tiab] OR intelligence [MeSH] OR language [tiab] OR language [MeSH] OR “problem solving” [tiab] OR “problem solving” [MeSH] OR hippocamp* [tiab] OR hippocamp* [MeSH] OR “executive function” [tiab] OR neuro* [tiab] OR neuro* [MeSH] OR “processing speed” [tiab] OR “processing speed” [MeSH] |
| #4 | #1 AND #2 AND #3 |
| #5 | "Diet Surveys"[Mesh] OR "Cohort Studies"[Mesh] OR cohort*[Tiab] OR prospective[Tiab] OR longitudinal[Tiab] |
| #6 | Randomized Controlled Trial[Publication Type] OR Controlled Clinical Trial[Publication Type] OR Pragmatic Clinical Trial[Publication Type] OR Clinical Study[Publication Type] OR Adaptive Clinical Trial[Publication Type] OR Equivalence Trial[Publication Type] OR Clinical Trial[Publication Type] OR Clinical Trial, Phase I[Publication Type] OR Clinical Trial, Phase II[Publication Type] OR Clinical Trial, Phase III[Publication Type] OR Clinical Trial, Phase IV[Publication Type] OR Clinical Trial Protocol[Publication Type] OR multicenter study[Publication Type] OR "Clinical Studies as Topic"[Mesh] OR "Clinical Trials as Topic"[Mesh] OR "Clinical Trial Protocols as Topic"[Mesh] OR "Multicenter Studies as Topic"[Mesh] OR "Random Allocation"[Mesh] OR "Double-Blind Method"[Mesh] OR "Single-Blind Method"[Mesh] OR "Placebos"[Mesh:NoExp] OR "Control Groups"[Mesh] OR "Cross-Over Studies"[Mesh] OR random*[Title/Abstract] OR sham[Title/Abstract] OR placebo*[Title/Abstract] OR ((singl*[Title/Abstract] OR doubl*[Title/Abstract]) AND (blind*[Title/Abstract] OR dumm*[Title/Abstract] OR mask*[Title/Abstract])) OR ((tripl*[Title/Abstract] OR trebl*[Title/Abstract]) AND (blind*[Title/Abstract] OR dumm*[Title/Abstract] OR mask*[Title/Abstract])) OR "control study"[tiab:~3] OR "control studies"[tiab:~3] OR "control group"[tiab:~3] OR "control groups"[tiab:~3] OR "healthy volunteers"[tiab:~3] OR "control trial"[tiab:~3] OR "control trials"[tiab:~3] OR "controlled study"[tiab:~3] OR "controlled trial"[tiab:~3] OR "controlled studies"[tiab:~3] OR "controlled trials"[tiab:~3] OR "clinical study"[tiab:~3] OR "clinical studies"[tiab:~3] OR "clinical trial"[tiab:~3] OR "clinical trials"[tiab:~3] OR Nonrandom*[Title/Abstract] OR non random*[Title/Abstract] OR non-random*[Title/Abstract] OR quasi-random*[Title/Abstract] OR quasirandom*[Title/Abstract] OR "phase study"[tiab:~3] OR "phase studies"[tiab:~3] OR "phase trial"[tiab:~3] OR "phase trials"[tiab:~3] OR "crossover study"[tiab:~3] OR "crossover studies"[tiab:~3] OR "crossover trial"[tiab:~3] OR "crossover trials"[tiab:~3] OR "cross-over study"[tiab:~3] OR "cross-over studies"[tiab:~3] OR "cross-over trial"[tiab:~3] OR "cross-over trials"[tiab:~3] OR ((multicent*[tiab] OR multi-cent*[tiab] OR open label[tiab] OR open-label[tiab] OR equivalence[tiab] OR superiority[tiab] OR non-inferiority[tiab] OR noninferiority[tiab] OR quasiexperimental[tiab] OR quasi-experimental[tiab]) AND (study[tiab] OR studies[tiab] OR trial*[tiab])) OR allocated[tiab] OR pragmatic study[tiab] OR pragmatic studies[tiab] OR pragmatic trial*[tiab] OR practical trial*[tiab] |
| #7 | "Epidemiologic Methods"[Mesh:NoExp] OR "Epidemiologic Studies"[Mesh] OR "Observational Studies as Topic"[Mesh] OR "Clinical Studies as Topic"[Mesh] OR "Single-Case Studies as Topic"[Mesh] OR "Organizational Case Studies"[Mesh] OR observational study[Publication Type] OR validation study[Publication Type] OR clinical study[Publication Type] OR case reports[Publication Type] OR "observational study"[tiab:~3] OR "observational studies"[tiab:~3] OR "observational design"[tiab:~3] OR "observational analysis"[tiab:~3] OR "observational analyses"[tiab:~3] OR ((cohort*[tiab] OR prospective[tiab] OR follow-up[tiab] OR longitudinal[tiab] OR long-term[tiab] OR retrospective[tiab]) AND (study[tiab] OR studies[tiab] OR design[tiab] OR analysis[tiab] OR analyses[tiab] OR data[tiab] OR review[tiab])) OR case control*[tiab] OR case comparison*[tiab] OR case-referent[tiab] OR "population study"[tiab:~3] OR "population studies"[tiab:~3] OR "population analysis"[tiab:~3] OR "population analyses"[tiab:~3] OR "descriptive study"[tiab:~3] OR "descriptive studies"[tiab:~3] OR "descriptive design"[tiab:~3] OR "descriptive analysis"[tiab:~3] OR "descriptive analyses"[tiab:~3] OR "multidimensional study"[tiab:~3] OR "multidimensional studies"[tiab:~3] OR "multidimensional design"[tiab:~3] OR "multidimensional analysis"[tiab:~3] OR "multidimensional analyses"[tiab:~3] OR "cross-sectional study"[tiab:~3] OR "cross-sectional studies"[tiab:~3] OR "cross-sectional design"[tiab:~3] OR "cross-sectional analysis"[tiab:~3] OR "cross-sectional analyses"[tiab:~3] OR "cross-sectional research"[tiab:~3] OR "cross-sectional survey"[tiab:~3] OR "cross-sectional findings"[tiab:~3] OR natural experiment*[tiab] OR quasi experiment*[tiab] OR "nonexperimental study"[tiab:~3] OR "nonexperimental studies"[tiab:~3] OR "nonexperimental design"[tiab:~3] OR "nonexperimental analysis"[tiab:~3] OR "nonexperimental analyses"[tiab:~3] OR "prevalence study"[tiab:~3] OR "prevalence studies"[tiab:~3] OR "prevalence analysis"[tiab:~3] OR "prevalence analyses"[tiab:~3] OR case series[tiab] OR "case report"[tiab:~3] OR "case reports"[tiab:~3] OR "case study"[tiab:~3] OR "case studies"[tiab:~3] OR "case histories"[tiab:~3] |

| | |
|---|---|
| #8 | "systematic review" |
| #9 | #5 OR #6 OR #7 OR #8 |
| #10 | #4 AND #9 |
| #11 | #10 NOT (("Child"[Mesh] OR "Infant"[Mesh] OR "Adolescent"[Mesh]) NOT "Adult"[Mesh]) |
| #12 | #11 NOT (("Animals"[Mesh] OR "Animal Experimentation"[Mesh] OR "Models, Animal"[Mesh] OR "Vertebrates"[Mesh]) NOT ("Humans"[Mesh] OR "Human Experimentation"[Mesh])) |
| #13 | #12 NOT ("Breast Feeding"[Majr] OR "Milk, Human"[Majr]) |
| #14 | #13 AND (English[Filter]) |
| #15 | #14 AND (("2023/08/31"[Date - Publication] : "2024/12/31"[Date - Publication])) |
| #16 | ((("Cardio*"[tiab] OR "Cancer"[tiab]) OR "Injury"[tiab] OR "Diabetes"[tiab] OR "Heart"[tiab])) " |
| #17 | #15 NOT #16 |
| | **Database: Scopus** |
| #1 | TITLE-ABS-KEY ((ferment* OR cultur* OR leaven*) W/6 (food* OR drink* OR beverage*) OR "starter culture*") OR TITLE-ABS-KEY ((ferment* OR cultur* OR leaven*) W/2 product*) OR TITLE-ABS-KEY (((ferment* OR culture* OR sour*) W/6 (milk OR dairy OR cream* OR quark)) OR buttermilk OR cheese* OR yoghurt OR yogurt OR yoghourt OR yakult OR kefir OR lassi OR kumis OR koumiss OR kajmak OR airag OR ayran OR calpis OR borhani OR chal OR doogh OR kvass OR skyr OR amasi OR bouza OR butter* OR chal OR filmjolk OR kishk OR labne*) OR TITLE-ABS-KEY (((Ferment* OR cultur* OR cured) W/6 (meat* OR fish* OR seafood* OR shellfish OR sausage*)) OR "salami" OR "pepperoni" OR peperoni OR "chorizo" OR "cervelat" OR "mettwurst" OR "summer sausage" OR "sucuk" OR "dried meat*" OR "dried sausage*" OR "dry sausage*" OR "fish sauce*" OR "shrimp paste" OR "shrimp sauce" OR "oyster sauce" OR "prosciutto" OR "pancetta" OR "saucisson" OR sucuk) OR TITLE-ABS-KEY (((Ferment* OR cultur*) W/6 (fruit* OR vegetable* OR coconut* OR almond* OR hazelnut* OR nut OR cucumber* OR lemon* OR citrus OR cabbage* OR cauliflower* OR pepper* OR carrot* OR olive* OR onion* OR sago)) OR "sauerkraut" OR "table olive*" OR pickle* OR "kimchi" OR "paocai" OR torshi) OR TITLE-ABS-KEY (((Ferment* OR cultur*) W/6 (tea OR teas OR juice*)) OR "beer" OR "wine" OR cider* OR shochu OR kombucha* OR "pulque" OR puer OR "pu-er*" OR "fuzhuan" OR "dark tea*" OR "yellow tea*" OR coffee OR shalgam OR hardaliye) OR TITLE-ABS-KEY (((Ferment* OR cultur*) W/6 (soy OR soya OR bean* OR pea OR peas OR lentil* OR chickpea* OR legume* OR pulse* OR poi)) OR "soy* sauce*" OR "soybean paste*" OR miso* OR tempeh* OR tempe OR "natto" OR "doenjang" OR "doubanjiang" OR douchi OR "gochujang" OR cheonggukjang OR tsukemono OR garri) OR TITLE-ABS-KEY (((Ferment* OR cultur* OR leaven*) W/6 (cereal* OR grain* OR wheat* OR oat OR oats OR rice* OR millet* OR sorghum* OR maize* OR rye OR barley* OR chia OR oilseed* OR teff)) OR "bread" OR "sourdough" OR "crispbread" OR "boza" OR "ogi" OR dosa OR "tarhana" OR "buckwheat" OR "spelt" OR "einkorn" OR "quinoa" OR "amaranth" OR "tef" OR "bushera" OR chica OR chicha OR choujiu OR injera OR mahewu OR ogiri OR pozol OR ugba) OR TITLE-ABS-KEY (((Ferment* OR cultur*) W/6 (condiment* OR relish* OR horseradish OR dressing* OR seasoning* OR sauce* OR cocoa* OR tuber OR "acetic acid")) OR chocolate* OR vinegar* OR "tabasco" OR "sriracha" OR "Worcestershire" OR "Worcester") |
| #2 | TITLE-ABS-KEY (((food OR *nutrient* OR eating OR nutrit*) W/6 (intake* OR habit* OR behavior* OR pattern* OR consumption OR suppl* OR ingestion)) OR diet* OR meal*) |
| #3 | TITLE-ABS-KEY (cognit*) OR INDEXTERMS (cognit*) OR TITLE-ABS-KEY (brain) OR INDEXTERMS (brain) OR TITLE-ABS-KEY (memory) OR INDEXTERMS (memory) OR TITLE-ABS-KEY (attention) OR INDEXTERMS (attention) OR TITLE-ABS-KEY (nerv*) OR INDEXTERMS (nerv*) OR TITLE-ABS-KEY (learning) OR INDEXTERMS (learning) OR TITLE-ABS-KEY (intelligence) OR INDEXTERMS (intelligence) OR TITLE-ABS-KEY (language) OR INDEXTERMS (language) OR TITLE-ABS-KEY ( "problem solving" ) OR INDEXTERMS ( "problem solving" ) |
| #4 | #1 AND #2 AND #3 *(add combination of string numbers to the field in advanced search in the field "Combined queries…")* |

| | |
|---|---|
| #5 | TITLE-ABS-KEY (random* OR sham OR placebo*) OR TITLE-ABS-KEY ((singl* OR doubl*) W/1 (blind* OR dumm* OR mask*)) OR TITLE-ABS-KEY ((tripl* OR trebl*) W/1 (blind* OR dumm* OR mask*)) OR TITLE-ABS-KEY (control* W/3 (study OR studies OR trial* OR group*)) OR TITLE-ABS-KEY (clinical W/3 (study OR studies OR trial*)) OR TITLE-ABS-KEY (Nonrandom* OR "non random*" OR non-random* OR quasi-random* OR quasirandom*) OR TITLE-ABS-KEY (phase W/3 (study OR studies OR trial*)) OR TITLE-ABS-KEY ((crossover OR cross-over) W/3 (study OR studies OR trial*)) OR TITLE-ABS-KEY ((multicent* OR multi-cent*) W/3 (study OR studies OR trial*)) OR TITLE-ABS (allocated) OR TITLE-ABS-KEY (("open label" OR open-label) W/5 (study OR studies OR trial*)) OR TITLE-ABS-KEY ((equivalence OR superiority OR non-inferiority OR noninferiority) W/3 (study OR studies OR trial*)) OR TITLE-ABS-KEY ("pragmatic study" OR "pragmatic studies") OR TITLE-ABS-KEY ((pragmatic OR practical) W/3 trial*) OR TITLE-ABS-KEY ((quasiexperimental OR quasi-experimental) W/3 (study OR studies OR trial*)) OR TITLE (trial) OR KEY (trial) |
| #6 | TITLE-ABS-KEY (observational W/3 (study OR studies OR design OR analysis OR analyses)) OR TITLE-ABS-KEY (cohort*) OR TITLE-ABS-KEY (prospective W/7 (study OR studies OR design OR analysis OR analyses)) OR TITLE-ABS-KEY (("follow up" OR followup) W/7 (study OR studies OR design OR analysis OR analyses)) OR TITLE-ABS-KEY ((longitudinal OR longterm OR (long W/1 term)) W/7 (study OR studies OR design OR analysis OR analyses OR data)) OR TITLE-ABS-KEY (retrospective W/7 (study OR studies OR design OR analysis OR analyses OR data OR review)) OR TITLE-ABS-KEY ((case W/1 control) OR (case W/1 comparison) OR (case W/1 controlled)) OR TITLE-ABS-KEY (case-referent W/3 (study OR studies OR design OR analysis OR analyses)) OR TITLE-ABS-KEY (population W/3 (study OR studies OR analysis OR analyses)) OR TITLE-ABS-KEY (descriptive W/3 (study OR studies OR design OR analysis OR analyses)) OR TITLE-ABS-KEY ((multidimensional OR (multi W/1 dimensional)) W/3 (study OR studies OR design OR analysis OR analyses)) OR TITLE-ABS-KEY (cross W/1 sectional W/7 (study OR studies OR design OR research OR analysis OR analyses OR survey OR findings)) OR TITLE-ABS-KEY ((natural W/1 experiment) OR (natural W/1 experiments)) OR TITLE-ABS-KEY (quasi W/1 (experiment OR experiments OR experimental)) OR TITLE-ABS-KEY (("non experiment" OR nonexperiment OR "non experimental" OR nonexperimental) W/3 (study OR studies OR design OR analysis OR analyses)) OR TITLE-ABS-KEY (prevalence W/3 (study OR studies OR analysis OR analyses)) OR TITLE-ABS-KEY ("case series") OR TITLE-ABS-KEY (case W/3 (report OR reports OR study OR studies OR histories)) |
| #7 | TITLE-ABS-KEY ("systematic review") |
| #8 | #5 OR #6 OR #7 *(add combination of string numbers to the field in advanced search in the field "Combined queries…")* |
| #9 | #4 AND #8 *(add combination of string numbers to the field in advanced search in the field "Combined queries…")* |
| #10 | (KEY (animal* OR nonhuman)) AND NOT (KEY (human*)) |
| #11 | #9 AND NOT #10 *(add combination of string numbers to the field in advanced search in the field "Combined queries…")* |
| #12 | (KEY (infant* OR child*)) AND NOT (KEY (adult* OR aged)) |
| #13 | #11 AND NOT #12 *(add combination of string numbers to the field in advanced search in the field "Combined queries…")* |
| | *Limit #13 to English using the language filter* |
| | *Limit #13 to 1970 - 2023 using the Year filter (Range from 1970 to 2023)* |
| | **Database: Cochrane** |
| #1* | ((ferment* OR cultur* OR leaven*) NEAR/6 (food* OR drink* OR beverage* OR product*) OR (starter NEXT culture*)) OR (((ferment* OR culture* OR sour*) NEAR/6 (milk OR dairy OR cream*)) OR buttermilk OR cheese* OR yoghurt OR yogurt OR yoghourt OR yakult OR quark OR kefir OR lassi OR kumis OR koumiss OR kajmak OR airag OR ayran OR calpis OR borhani OR chal OR doogh OR kvass OR skyr OR amasi OR bouza OR butter* OR chal OR filmjolk OR kishk OR labne*) OR (((Ferment* OR cultur* OR cured) NEAR/6 (meat* OR fish* OR seafood* OR shellfish OR sausage*)) OR "salami" OR "pepperoni" OR peperoni OR "chorizo" OR "cervelat" OR "mettwurst" OR "summer sausage" OR "sucuk" OR (dried NEXT meat*) OR (dried NEXT sausage*) OR (dry NEXT sausage*) OR (fish NEXT sauce*) OR "shrimp paste" OR "shrimp sauce" OR "oyster sauce" OR "prosciutto" OR "pancetta" OR "saucisson" OR sucuk) OR (((Ferment* OR cultur*) NEAR/6 (fruit* OR vegetable* OR coconut* OR almond* OR hazelnut* OR nut OR cucumber* OR lemon* OR citrus OR cabbage* OR cauliflower* OR pepper* OR carrot* OR olive* OR onion* OR sago)) OR "sauerkraut" OR (table NEXT olive*) OR pickle* OR "kimchi" OR "paocai" OR torshi) OR (((Ferment* OR cultur*) NEAR/6 (tea OR teas OR juice*)) OR "beer" OR "wine" |

| | |
|---|---|
| | OR cider* OR shochu OR kombucha* OR "pulque" OR puer OR pu-er* OR "fuzhuan" OR (dark NEXT tea*) OR (yellow NEXT tea*) OR coffee OR shalgam OR hardaliye) OR (((Ferment* OR cultur*) NEAR/6 (soy OR soya OR bean* OR pea OR peas OR lentil* OR chickpea* OR legume* OR pulse* OR poi)) OR (soy* NEXT sauce*) OR (soybean NEXT paste*) OR miso* OR tempeh* OR tempe OR "natto" OR "doenjang" OR "doubanjiang" OR douchi OR "gochujang" OR cheonggukjang OR tsukemono OR garri) OR (((Ferment* OR cultur* OR leaven*) NEAR/6 (cereal* OR grain* OR wheat* OR oat OR oats OR rice* OR millet* OR sorghum* OR maize* OR rye OR barley* OR chia OR oilseed* OR teff)) OR "bread" OR "sourdough" OR "crispbread" OR "boza" OR "ogi" OR dosa OR "tarhana" OR "buckwheat" OR "spelt" OR "einkorn" OR "quinoa" OR "amaranth" OR "tef" OR "bushera" OR chica OR chicha OR choujiu OR injera OR mahewu OR ogiri OR pozol OR ugba) OR (((Ferment* OR cultur*) NEAR/6 (condiment* OR relish* OR horseradish OR dressing* OR seasoning* OR sauce* OR cocoa* OR tuber OR "acetic acid")) OR chocolate* OR vinegar* OR "tabasco" OR "sriracha" OR "Worcestershire" OR "Worcester") |
| #2* | (((food OR macronutrient* OR eating) NEAR/6 (intake* OR habit* OR behavior* OR pattern*)) OR diet* OR intake OR ingestion OR suppl* OR consumption OR meal* OR nutrient* OR nutrit*) |
| #3* | (cognit* AND (impair* OR decline) OR (memory OR brain OR attention OR nerv* OR learning OR intelligence OR language OR "problem solving" OR hippocamp* OR "executive function" OR neuro* OR "processing speed")) |
| #4* | #1 AND #2 AND #3 |
| ** | Choose in the field "Limits" the following options: (a) Content type: "Cochrane Reviews" and "Trials"; (b) Date published on the Cochrane Library: Between "January 1970" and "August 2023" |

**Supplementary table S2.** Data extraction table

| |
|---|
| Name of persons extracting data |
| Specifics about the paper/study |
| Author, year |
| Number from CADIMA |
| Title of the paper (with DOI of the paper/link) |
| Study name/cohort |
| Name of the other papers from the same study/cohort |
| Number(s) from CADIMA for this/those other paper(s) |
| Clinical trial ID/study registration ID (Link) |
| Type of study/study design |
| Other sources of information for this study? |
| Country |
| Any errata, corrected re-publication or retraction identified for the publication? |
| Aim/objective of study |
| Population characterization |
| Population/subjects inclusion/exclusion criteria |
| Population/subjects intervention (age, race, number of participants in the group starting and finishing the trial, gender %) |

| |
|---|
| Population/subjects control (age, race, number of participants in the group starting and finishing the trial, gender %) |
| Intervention/product characterization |
| Raw material and processing information for intervention product (with as more info as you can find) |
| Intervention product fermented? |
| Intervention product fermented with *Lactobacillus* spp. and *Bifidobacteria* spp.? As much info as you can find |
| Comparator/Control characterization |
| Raw material and processing information for comparator product (all information found, text/table) |
| Comparator product indistinguishable to intervention product in matrix, colour and taste? |
| Comparator product fermented? |
| Comparator product fermented with *Lactobacillus* spp. and *Bifidobacteria* spp.? |
| Intervention |
| Dose/Frequency/Duration Intervention |
| Dose/Frequency/Duration Comparator/Control |
| Follow-up time (better definition of follow up) |
| Outcome |
| Method for cognitive measurement (all info provided in the paper) |
| Outcome (no p values needed) |
| Secondary Outcome (any secondary outcome noted in the results) |
| Effect (+/-/none) |
| Main findings (results in detail, with p values) |
| Mechanism of action |
| Any information on mechanism of action in this paper? |
| Given information on mechanism of action |
| The papers from the same authors with info about product characteristic/mechanism of action (if found by chance) |
| Quality and Bias, Yes or No? |

**Supplementary table S3.** Cohorts and geographical region

| Cohort Name | Articles Using the Cohort | Notes on Overlap / Focus |
|---|---|---|
| NHANES | Park et al. (2013), Han et al. (2024), Park et al. (2019) | Same cohort; different cycles: 1988–1994 (Park 2013), 2011–2014 (Han 2024), 1999–2002 (Park 2019) |
| Otassha Study | Kim et al. (2023), Suzuki et al. (2024) | Same cohort; Kim used broader sample, Suzuki focused on older women |

| | | |
|---|---|---|
| PREDIMED-Plus | Ni et al. (2022), Muñoz-Garach et al. (2021) | Same cohort; potentially different subpopulations or analysis focuses |
| KIHD (Kuopio Ischaemic Heart Disease Study) | Ylilauri et al. (2022) | Independent cohort |
| SU.VI.MAX / SU.VI.MAX 2 | Kesse-Guyot et al. (2016) | Independent cohort; includes follow-up SU.VI.MAX 2 |
| CLSA | Tessier et al. (2021) | Independent cohort |
| B-PROOF Study | de Goeij et al. (2020) | Independent cohort |
| CoLaus\|PsyCoLaus | Ortega et al. (2024) | Independent cohort |
| Indonesian Rural/Urban Samples | Hogervorst et al. (2008), Hogervorst et al. (2011) | Possibly overlapping populations in rural and urban areas |

B-PROOF: B-vitamins for the PRevention of Osteoporotic Fractures; CLSA: Canadian Longitudinal Study on Aging; CoLaus|PsyCoLaus: Cohorte Lausannoise | Psychiatric arm of CoLaus; NHANES: National Health and Nutrition Examination Survey; PREDIMED-Plus: PREvención con DIeta MEDiterránea (in English Prevention with Mediterranean Diet)

**Supplementary table S4.** Confounding factors in interventional studies

| **Reference** | **Confounding Factors** |
|---|---|
| Benton et al., 2007 | Cognitive status, mental and systematic health; Psychological/neurological issues, age |
| Cannavale et al., 2022 | Psychological/neurological issues, gastrointestinal illness, age |
| Chung et al., 2014 | Cognitive status, BMI, gastrointestinal illness, recent supplement use; Psychological/neurological issues, age |
| Handajani et al., 2020 | Diabetes, other fermented foods, MCI included |
| Hwang et al., 2019 | Cognitive status, Psychological/neurological issues; use of nootropics or cognitive-enhancing supplements; age |
| Ohsawa et al., 2018 | Cognitive status |
| Reid et al., 2018 | Lifestyle: Abstained from regular exercise; age |

BMI: Body Mass Index; MCI: Mild Cognitive Impairment

**Supplementary table S5.** Health-Related Factors in observational studies

| Study | Medication | Diabetes | BP | Lipids | CVD | Depression | Eating Disorders |
|---|---|---|---|---|---|---|---|
| Ylilauri et al. (2022) | Yes | Yes | Yes | Yes | Yes | No | No |
| Ni et al. (2022) | Yes | Yes | Yes | Yes | No | Yes | Yes |
| Muñoz-Garach et al. (2021) | Yes | Yes | Yes | Yes | No | Yes | No |
| Tessier et al. (2021) | No | Yes | No | No | Yes | Yes | No |
| Kesse-Guyot et al. (2016) | Yes | Yes | Yes | No | Yes | Yes | No |
| Ortega et al. (2024) | Yes | Yes | Yes | No | Yes | Yes | No |
| Suzuki et al. (2024) | No | Yes | No | Yes | No | Yes | No |
| Kim et al. (2023) | No | Yes | Yes | Yes | No | No | No |
| De Goeij et al. (2020) | No | Yes | No | No | No | No | No |
| Park et al. (2013) | No | No | No | No | No | No | No |
| Hogervorst et al. (2008) | * | No | No | No | No | * | No |
| Hogervorst et al. (2011) | * | No | No | No | No | * | No |

BP: Blood pressure; CVD: Cardiovascular disease

**Supplementary table S6.** Lifestyle associated factors in observational studies

| Study | Alcohol Consumption | Smoking | Physical Activity | Social Activities | Nutrition / Energy Intake | Other Food Groups |
|---|---|---|---|---|---|---|
| Ylilauri et al. (2022) | Yes | Yes | Yes | No | Yes | Yes |
| Ni et al. (2022) | Yes | Yes | Yes | No | Yes | Yes |
| Muñoz-Garach et al. (2021) | Yes | Yes | Yes | No | Yes | Yes |
| Tessier et al. (2021) | Yes | Yes | Yes | Yes | Yes | No |
| Kesse-Guyot et al. (2016) | Yes | Yes | Yes | No | Yes | Yes |
| Ortega et al. (2024) | Yes | Yes | Yes | No | Yes | Yes |
| Suzuki et al. (2024) | No | No | Yes | No | No | Yes |
| Kim et al. (2023) | No | No | No | No | No | Yes |
| De Goeij et al. (2020) | Yes | Yes | Yes | No | Yes | Yes |
| Park et al. (2013) | No | Yes | Partial (covariate*) | No | Yes | No |

| | | | | | | |
|---|---|---|---|---|---|---|
| Hogervorst et al. (2008) | No | No | No | No | No | Yes |
| Hogervorst et al. (2011) | No | No | No | No | No | No |

**Supplementary table S7.** The effect of dairy- and plant-based fermented food on different cognitive domains

| Type of Fermented Food | Cognitive Domain | Reference |
|---|---|---|
| **Dairy-based** | Attention | Ohsawa et al., 2018; Park et al., 2019; Han et al., 2024; Park et al., 2013 |
| | Memory (delayed, verbal, recall) | Ohsawa et al., 2018; Cannavale et al., 2022; Chung et al., 2014; Tessier et al., 2021; Ni et al., 2022 |
| | Executive function | Chung et al., 2014; Tessier et al., 2021; de Goeij et al., 2020; Han et al., 2024 |
| | Processing speed | Park et al., 2013; Han et al., 2024; de Goeij et al., 2020 |
| | Information processing speed | Park et al., 2013; de Goeij et al., 2020 |
| | Verbal fluency | Han et al., 2024; Ni et al., 2022; Tessier et al., 2021 |
| | Global cognition | Muñoz-Garach et al., 2021; Han et al., 2024; Kim et al., 2023; Suzuki et al., 2024 |
| | Risk of dementia | Ylilauri et al., 2022; Kim et al., 2023; Suzuki et al., 2024; Ortega et al., 2024 |
| | Visuomotor/Visual memory | Ylilauri et al., 2022; Park et al., 2013 |
| **Plant-based** | Memory (verbal learning, recall) | Reid et al., 2018; Hwang et al., 2019; Hogervorst et al., 2008; Hogervorst et al., 2011 |
| | Attention | Hwang et al., 2019 |
| | Language (semantic, naming) | Handajani et al., 2020 |
| | Protection against cognitive impairment | Reid et al., 2018 |

**Supplementary text for section: 4.2.** Substantiation of a causal relationship between consumption of the fermented food and the functional effect

4.2.2 "*Magnitude of the effect and its physiological relevance*"

Among RCTs that utilized validated cognitive tools, several studies reported statistically significant effects in specific domains. For example, **Ohsawa et al. (2018)** found that healthy middle-aged adults who consumed fermented milk for eight weeks exhibited statistically significant improvements in RBANS total score, attention, and delayed memory scores. The attention score improved significantly both within the intervention group ($p = 0.006$) and in comparison to the placebo at week 8 ($p = 0.028$), indicating a robust and meaningful effect. The total score and the delayed memory score also improved significantly within the group,

although the between-group difference was not statistically significant. These enhancements, especially in attention and delayed memory, alongside better scores on the coding subtest, suggest potential benefits in learning and memory performance, although not all effects were confirmed between groups. Similarly, **Hwang et al. (2019)** found that individuals with mild cognitive impairment who consumed a *Lactobacillus plantarum* C29-fermented soybean supplement for 12 weeks experienced statistically significant improvements in overall cognitive performance, particularly in the attention domain. Compared to the placebo group, the intervention group showed significantly greater gains in overall cognition ($z = 2.36$, $p = 0.02$), with the most pronounced improvements observed in attention ($z = 2.34$, $p = 0.02$). These cognitive enhancements were accompanied by increased levels of brain-derived neurotrophic factor (BDNF), underscoring both the statistical significance and physiological relevance of the findings. Handajani et al. (2020) found that Tempeh A and B consumption led to improvements in global cognition among older adults with mild cognitive impairment, as measured by MMSE. Specifically, Tempeh A improved MMSE scores from $19.7 \pm 5.4$ to $22.4 \pm 4.8$ ($p < 0.001$), while Tempeh B improved scores from $19.6 \pm 5.0$ to $23.7 \pm 3.9$ ($p < 0.001$), compared to no significant change in the control group ($24.3 \pm 1.34$ to $24.4 \pm 1.3$, $p = 0.564$). Notably, only Tempeh A, which had a lower microbial count, also led to a significant improvement in the language domain as assessed by the Boston Naming Test (BNT), increasing scores from $11.4 \pm 2.6$ to $12.3 \pm 2.5$ ($p = 0.003$), while no such improvement was observed in the other groups. This suggests a possible domain-specific physiological relevance of microbial composition in fermented foods.

In a study with a more focused design, **Chung et al. (2014)** observed statistically significant improvements in attention and working memory following 12 weeks of fermented milk consumption in healthy older adults. Specifically, the Digit Span Test (DST) total score was significantly higher in the intervention group compared to the placebo group ($p = 0.043$), while within-group improvements were also observed in DST-forward, DST-backward, and K-MMSE scores. However, no significant between-group differences were found for K-MMSE, Word List Memory, or Word List Recall tests. These improvements, while observed on well-established cognitive tests, the overall study was described by the authors as a pilot one with a relatively small sample size, and they did not benchmark the magnitude of change against clinically meaningful thresholds or long-term cognitive outcomes. Moreover, improvements were not observed consistently across all neuropsychological tests, and there was no effect on biomarkers like BDNF and WBV, which were explored as possible mechanisms. Moving to studies with more domain-specific outcomes, **Reid et al. (2018)** reported that participants who consumed fermented *Laminaria japonica* daily for six weeks showed significant improvements in cognitive performance and specifically for short-term memory. Specifically, greater improvements were observed in the K-MMSE, numerical memory, and iconic memory tests compared to the control group. These results indicate enhanced short-term working memory and visual sensory memory. However, the study did not assess broader cognitive domains, making it unclear how these improvements might generalize to overall cognitive functioning.

In contrast, not all studies showed consistent or clinically meaningful improvements. For instance, Park et al. (2019) examined the impact of *Saccharina japonica* fermented with *Lactobacillus* and observed better performance in certain memory tasks. However, no statistically significant differences were found between the FSJ and placebo groups across multiple validated cognitive tests (e.g., Digit Span, Digit Symbol Coding, Block Design, Operation-Word Span, Raven's Test). As the improvements were based only on within-group comparisons and not supported by between-group differences, their clinical relevance remains limited. Benton **et al. (2007)** found no consistent cognitive benefit from the intervention, while the participants who consumed the probiotic drink demonstrated slightly poorer performance on some memory tasks in comparison to the placebo group. These differences, although they were statistically significant, were small in magnitude and not consistently observed across all tests. The authors themselves concluded that the results did not support the idea that this probiotic drink improved cognitive performance. Similarly, Cannavale et al. (2022) investigated a kefir-based dairy drink and observed improvements in sustained attention and reaction time within the intervention group. However, these differences were not statistically significant compared to the control group. The short study duration and absence of long-term follow-up further limited conclusions regarding physiological relevance.

Across the observational studies investigating fermented food consumption and cognitive outcomes, the magnitude of the effect is generally unknown, and in nearly all cases, the findings have not been demonstrated to be physiologically relevant. Studies, such as **Tessier et al. (2021), Ylilauri et al. (2022)**, **Kim et al. (2023), Han et al. (2024)** and **Suzuki et al. (2024),** reported positive associations between fermented foods (cheese, yogurt, tempeh) using standardized cognitive assessments such as the MMSE, Trail Making Test, Verbal Fluency Test, or Hopkins Verbal Learning Test (HVLT). Among them, only Tessier et al. (2021) and Suzuki et al. (2024) provided comparative results between high and low consumer groups, offering some insight into dose-dependent trends. However, none of these studies benchmarked the observed changes against established clinical thresholds, limiting the ability to determine whether the reported cognitive improvements were meaningful in terms of daily functioning or long-term health outcomes. Nonetheless, some observational findings provide indirect support for the physiological relevance of fermented food consumption. For instance, Ylilauri et al. (2022) showed that participants in the highest quartile of cheese intake had a significantly lower risk of dementia over a 22-year follow-up. Similarly, Ortega et al. (2024), which clearly differentiated fermented from non-fermented dairy, found favorable associations between fermented dairy intake and cognitive outcomes. These findings are suggestive of a possible protective effect that aligns with the modest but statistically significant improvements seen in RCTs. Moreover, the use of stratified intake categories (e.g., quartiles in Ni et al. (2022) and Ylilauri et al. (2022)) allows for the identification of dose-response patterns, although most studies lacked precise intake quantification or microbial strain verification.

Other studies, such as **Hogervorst et al. (2008, 2011)**, also suggested possible cognitive benefits of fermented soy or dairy products but similarly lacked clear quantification of effect magnitude or clinical benchmarking. Conversely, several studies either failed to isolate fermented from non-fermented foods (Park et al., 2013; Muñoz-Garach et al., 2021) or incorporated fermented food consumption within broader dietary patterns (de Goeij et al., 2020; Kesse-Guyot et al., 2016), thereby diluting their ability to provide fermented food-specific insights. Furthermore, Ni et al. (2022) and Park et al. (2013) reported null or negative associations, which may stem from limited fermented food intake, insufficient follow-up duration, or lack of stratification by microbial quality.

*Duration range and physiological effect*

The duration of human studies analysed in this systematic review ranged from 20 days to over two years *(Ni et al., 2022; Benton et al., 2007; Kesse-Guyot et al., 2016; de Goeij et al., 2020; Ortega et al., 2024*).

Evidence suggests that probiotics can alter the gut microbiota and, through the gut-brain axis, impact cognitive function within a matter of weeks (*Kumar et al., 2024; Fekete et al., 2024*).

Fermented dairy products have been shown to produce noticeable cognitive benefits after four weeks of regular intake, with further enhancements observed at the eight-week mark in certain cognitive domains (*Tillisch et al., 2013; Cannavale et al., 2020; Cannavale et al., 2022; Chung et al., 2014; Ohsawa et al., 2018*).

The time frame for cognitive effects of fermented seaweed products varies. Acute intake of brown seaweed extract was found to enhance attention and reaction time within hours (*Haskell-Ramsay et al., 2018*). Two randomized, double-blind, placebo-controlled trials included in this review showed cognitive improvements from fermented seaweed products after several weeks of daily intake. Specifically, fermented *Laminaria japonica* enhanced short-term memory after six weeks (*Reid et al., 2018*), while fermented *Saccharina japonica* improved working memory and attention after eight weeks (*Park et al., 2019*). Emerging evidence also indicates that fermented soy products may improve cognitive function within relatively short durations. While many studies on fermented soy focus on animal models, human studies included in this review found cognitive improvements in older adults after six months of tempeh consumption (*Handajani et al., 2020*) while *L. plantarum*-fermented soybean (DW2009) showed cognitive benefits after only 12 weeks (*Hwang et al., 2019*). This relatively short timeframe may be attributed to the product’s composition, in addition to the 62.5% fermented soybean powder they added 37.5% freeze-dried *L. plantarum*, possibly the reason behind the higher baseline lactobacilli levels that were observed in the participants' gut microbiota.

Diary based RCT studies showed that longer-term consumption (eight to twelve weeks) of *Lactobacillus helveticus*–fermented milk drinks improved attention and delayed memory in both healthy middle-aged and senior adults (*Ohsawa et al., 2018; Chung et al., 2014*). Additionally, consumption of kefir improved relational memory of healthy adults aged 25-45, after four weeks (*Cannavale et al., 2022*). Conversely, consumption of a probiotic-containing milk drink, for only 20 days, was associated with slight impairments in episodic and long-term memory of adults aged 48-79 years (*Benton et al., 2007*).

Obs that examined the effects of tempeh, also showed its positive effects on cognitive function. Only one month of tempeh consumption improved memory performance, including immediate and delayed recall, related to learning ability in elderly Indonesian adults (*Hogervorst et al., 2011*).

Research on the cognitive impact of yogurt is limited, and intervention studies assessing the time required for yogurt to affect cognition are scarce. Cross-sectional analyses of the U.S. NHANES population found that older yogurt consumers scored higher in executive function and verbal fluency compared to non-consumers. However, due to the observational nature of these studies, they do not establish causality or clarify the duration needed for yogurt to have an effect (*Han et al., 2024;*). Similar case is with observational studies that examined the effects of cheese consumption, these studies did not confirm a causal relationship or specify the timeframe required for cheese to influence cognition (*Tessier et al., 2021; Park et al., 2013; Kim et al., 2023; Suzuki et al., 2024; de Goeij et al., 2020*). One long-term observational study found that cheese consumption reduced dementia incidence in men aged 42–60 years over a 22-year follow-up (*Ylilauri et al., 2022*). Meanwhile, one observational study showed that adding fermented dairy to the diet showed negligible and inconsistent effects on cognitive outcomes, after 5.6 years of dairy intake (*Ortega et al., 2024*).

## Supplementary Workflows

*Workflow: Mechanism of action (EFSA step 6)*

Is/are the mechanism/s by which the fermented food could exert the functional effect known?

In case several constituents are mediating the functionality, how does each constituent contribute to the functional effect?

Others points?

Conclude this section by narratively summarizing the main evidence and gaps

*Workflow: Bioavailability of bioactive compounds (EFSA step 2)*

Is/are the bioactive compound/s (molecule/s, microbe/s) known?

Where applicable, concisely summarize the relevant data and rationale to support that the fermented food for which the health claim is made is in a form for which the bioactive compounds (molecule, bacteria) is/are available to be used by the human body.

If available, describe any factors (e.g., formulation and processing) that could affect the absorption or utilization in the body of the bioactive entity in the fermented food for which the health claim is made.

Others points?  Conclude this section by narratively summarizing the main evidence and gaps

*Workflow: Characterization of the fermented foods and their bioactive compounds (EFSA step 2)*

Were specific, fermented foods investigated or fermented food as a whole? Was there an over-representation of specific fermented food or food group?

Were the fermented food and their bioactive compounds sufficiently characterized concerning

(i) the sources and specification of material used for the clinical studies,

(ii) the batch-to-batch variability in case multiple batches were used for the studies,

(iii) the analytical methods used to characterize the fermented food and their bioactive compounds used for the studies,

(iv) the quality system in place to ensure the quality of the fermented food and their bioactive compounds,

(v) others?

Were the fermented foods and their bioactive compounds sufficiently characterized concerning the manufacturing process (e.g., technology) to ensure its quality for human studies?

Were the fermented foods and their bioactive compounds sufficiently characterized concerning the manufacturing process to ensure its stability during the human studies (storage conditions, shelf-life)?

Sensory properties. Were the test and control foods sensory acceptable? Was there any information on the changes in food matrix if they were sensorial undesirable?

Conclude this section by narratively summarizing the main evidence and gaps

*Workflow: Safety*

Were adverse effects reported in these studies? What were these adverse effects? How many studies reported them? How is the fermentation (process) of these foods associated with the observed adverse effects?

Are populations identified who should avoid the fermented food?

Should a warning be made if the fermented food is consumed in excess?

Are there other restrictions with regard to the safety of the fermented food?

Conclude this section by narratively summarizing the main evidence and gaps.
